# Exopolysaccharides from *Enterococcus faecium* and *Streptococcus thermophilus*: Bioactivities, gut microbiome effects, and fermented milk rheology

**DOI:** 10.1016/j.fochx.2023.101073

**Published:** 2023-12-15

**Authors:** Mohammed Tarique, Abdelmoneim H. Ali, Jaleel Kizhakkayil, Shao-Quan Liu, Fatih Oz, Enes Dertli, Afaf Kamal-Eldin, Mutamed Ayyash

**Affiliations:** a Department of Food Science, College of Agriculture and Veterinary Medicine, United Arab Emirates University (UAEU), Al Ain, United Arab Emirates; b Department of Food Science, Faculty of Agriculture, Zagazig University, Zagazig 44511, Egypt; c Department of Nutrition and Health Sciences, College of Medicine and Health Sciences, United Arab Emirates University (UAEU), Al Ain, United Arab Emirates; d Department of Food Science and Technology, Faculty of Science, National University of Singapore, Science Drive 2, Singapore 117542, Singapore; e Department of Food Engineering, Faculty of Agriculture, Ataturk University, Erzurum 25240, Turkey; f Department of Food Engineering, Faculty of Chemical and Metallurgical Engineering, Yildiz Technical University, İstanbul, Turkey

**Keywords:** Exopolysaccharides, Bioactive functions, Prebiotics, Gut Microbiome

## Abstract

Exopolysaccharides (EPSs) are carbohydrate polymers that can be produced from probiotic bacteria. This study characterized the EPSs from *Enterococcus faecium* (EPS-LB13) and *Streptococcus thermophilus* (EPS-MLB10) and evaluated their biological and technological potential. The EPSs had high molecular weight and different monosaccharide compositions. The EPSs exhibited various biological activities at 250 mg/L, such as scavenging free radicals (10 % to 88.8 %), enhancing antioxidant capacity (714 to 2848 µg/mL), inhibiting pathogens (53 % to 74 %), and suppressing enzymes and cancer cells (2 % to 83 %), etc. The EPSs supported the growth of beneficial gut bacteria from Proteobacteria, Bacteroidetes, Firmicutes, and Acinetobacter in fecal fermentation with total Short-chain fatty acids production from 5548 to 6023 PPM. Moreover, the EPSs reduced the gelation time of fermented skimmed bovine milk by more than half. These results suggest that the EPSs from LB13 and MLB10 have promising applications in the dairy and pharmaceutical industries.

## Highlights

• EPS-LB13 and EPS-MLB13 showed good antioxidant and ACE-inhibition rates.
• EPSs exhibited activity against foodborne pathogens and antiproliferative effects on cancer cell lines.
• EPSs promoted selective bacteria (*Faecalibacterium prausnitzii*, *Ruminococcus bromii*) responsible for carbohydrate metabolism and short-chain fatty acid production.
• EPS-producing bacteria improved rheological properties of bovine skimmed milk.

## 1 Introduction

Exopolysaccharides (EPSs) are carbohydrate polymers produced by microorganisms that can be either tightly bound to the cell as a capsule layer or loosely attached to the cell surface which can be found in the media (Ayyash et al., 2020). Structurally, two types of EPS can be produced: homopolysaccharides and heteropolysaccharides, the former consisting of repeating units of one type of monosaccharide and the latter consisting of repeating units of multiple monosaccharides. EPSs are produced to protect microorganisms from environmental stress and enhance their adhesion to surfaces, and technologically, EPS confer rheological and sensory properties to fermented foods (Ayyash et al., 2020). Importantly, EPS are considered as parts of postbiotics which are the metabolites of mainly probiotics as well as other important microbial groups, that have beneficial effects on the host, such as anti-inflammatory, immunomodulatory, anti-proliferative, and antioxidant activities (Teame et al., 2020). Regarding the postbiotic function of EPS, more comprehensive studies are required to test both the bioactive and technological roles of EPS together with their physicochemical characteristics (Angelin and Kavitha, 2020, Ayyash et al., 2020).

Lactic acid bacteria (LAB) are one of the most important microbial groups for food industry as well as for probiotic applications and LABs were shown to produce distinct EPS at different levels which was demonstrated to be affected by various factors including strain-specific conditions, carbon sources, and environmental stress. Among LABs, *Enterococcus faecium* and *Streptococcus thermophilus* are two important species that are widely used in the fermented food industry and have been reported to have various *in vitro* health-promoting properties (Abarquero et al., 2022, Angelin and Kavitha, 2020). Additionally, *E. faecium* and *S. thermophilus* strains were reported to produce EPS with different structural properties as well as bioactive roles (Al-Nabulsi et al., 2022, Yang et al., 2023). The bioactive roles of EPS include modulation of the intestinal microbiota, and anti-cholesterol, anti-diabetic, antioxidant, antimicrobial, anti-inflammatory, and immunomodulatory activities (Abarquero et al., 2022, Al-Nabulsi et al., 2022, Angelin and Kavitha, 2020, Ayyash et al., 2020).

The health benefits of EPS from *E. faecium* and *S. thermophilus* may be attributed to their interactions with the gut microbiome, which is a complex ecosystem of microorganisms that plays a crucial role in maintaining host metabolic and immune homeostasis (Angelin and Kavitha, 2020, Yang et al., 2023). EPS may act as prebiotics, “substrates that are selectively utilized by host microorganisms conferring a health benefit” (Bello, Walter, Hertel, & Hammes, 2001). EPS may stimulate the growth and activity of beneficial bacteria, such as Bifidobacteria and Lactobacilli, and inhibit the growth of pathogenic bacteria, such as *E. coli* and *Salmonella* Typhimurium (Al-Nabulsi et al., 2022, Angelin and Kavitha, 2020, Ayyash et al., 2020). EPS was also shown to play roles in the regulation of intestinal health by acting as prebiotic (Pan, Han, & Zhou, 2020). Additionally, EPS may also modulate gut microbiome composition and function by influencing the production of short-chain fatty acids (SCFAs), which are important metabolites in the host's energy metabolism, intestinal barrier function, immune regulation, and inflammation: acetate, propionate, and butyrate (Angelin and Kavitha, 2020, Teame et al., 2020, Yang et al., 2023).

In addition to their health benefits, EPS from *E. faecium* and *S. thermophilus* may have technological benefits in food processing, especially for dairy products. EPS may influence the rheological properties of milk by increasing its viscosity, stability, creaminess, and mouthfeel (Abarquero et al., 2022). EPS may also improve the texture and appearance of fermented dairy products by reducing syneresis (whey separation), enhancing water retention, and forming stable gels (Abarquero et al., 2022, Tiwari et al., 2021). Therefore, EPS produced by *E. faecium* and *S. thermophilus* may be used as natural thickeners or stabilizers for dairy products.

Both functional and technological roles of EPS can be dependent on the structure of the produced EPS in which strain-specific conditions play crucial roles and more studies are required to determine the EPS production in different strains. Importantly there is also a lack of knowledge for testing the EPS produced by distinct strains for their dual activities in terms of bioactive and technological functions. From these perspectives, this study aimed to determine the EPS production characteristics of *E. faecium* (EPS-LB13) and *S. thermophilus* (EPS-MLB10) as dairy isolates. For this, EPS-LB13 and EPS-MLB10 were extracted from the correspondent strain and characterized in terms of monosaccharide composition, molecular weight, functional groups within its structure, zeta potential, and particle size. In terms of functional and bioactive roles, both EPSs were tested for their antioxidant, antimicrobial, and antibiofilm activities as well as prebiotic potentials that were determined by testing the probiotic strain-promoting effects, *in vitro* fecal fermentation and colon microbiota analysis. Finally, the technological functions of both EPSs were determined using a fermented skimmed bovine milk model.

## 2 Materials and methods

### 2.1 Bacterial propagation

The strains *E. faecium* MW725386 and *S. thermophilus* MW725391, which produced EPS-LB13 and EPS-MLB10, respectively, were previously isolated from traditional yogurt-like products (Labaneh) and characterized for their probiotic potentials. The strains were preserved at −80 °C and deposited in the GenBank (accession no. MW725386 & MW725391) (Tarique et al., 2022). The cultures were reactivated in de Man, Rogosa and Sharpe (MRS) broth at 37 °C for 24 h. All chemicals used in this study were purchased from Sigma-Aldrich (St. Louis, USA) unless otherwise specified.

### 2.2 EPS extraction and purification

Briefly, cultured bacteria were inoculated (1 %, v/v) in fresh sterile MRS broth supplemented with 20 g/L sucrose in a fermenter tank. The fermentation was carried out at 43 °C for 48 h maintaining pH 6 using sterile sodium hydroxide (NaOH) in fermenter (Biostream International, Netherlands). Biomass was discarded after centrifugation at 4000 RPM for 15 mins at 4 °C and the supernatant was mixed with 80 % trichloroacetic acid (TCA) to make up to 14 % TCA and stored for 1 h at room temperature. The supernatant was collected after centrifugation at 4000 RPM for 15 mins at 4 °C and mixed with two volumes of absolute chilled ethanol and stored for 24 h, at 4 °C. After centrifugation at 4000 RPM for 15 mins at 4 °C, the supernatant was discarded, and the precipitate was dissolved in warm deionized water. For the purification, the mixture was poured in 20 kDa MWCO Slide-A-Lyzer G2 dialysis cassette (Thermo Fisher Scientific, USA) and dialyzed against deionized water for 72 h at 4 °C. The EPS yield was calculated using the phenol–sulfuric acid method (Dubois, Gilles, Hamilton, Rebers, & Smith, 1956). A part of the purified water-soluble EPS-LB13 and EPS-MLB10 was used for the bioactivity tests, and the rest was freeze-dried and stored at −20 °C for further analysis.

### 2.3 Characterization of EPS-LB13 and EPS-MLB10

#### 2.3.1 Determination of average molecular mass and monosaccharide composition

The molecular weights (Mw) of the EPS-LB13 and EPS-MLB10 were determined by gel permeation chromatography (GPC) as described by Al-Nabulsi et al. (2022). Briefly, the samples were filtered through 0.22 μm syringe filters and injected in SIL-20AC autosampler of the Shimadzu HPLC system (Japan) equipped with a refractive index detector (RID-20A). Shim-pack GPC-802 column at 40 °C was used for the detection, and the samples were eluted with deionized water at a flow rate of 1 mL/min. Mw was calculated using a calibration curve (Fig. S1a) obtained with various pullulan standards (Mw = 800, 400, 200, 110, 50, 22, 10, 6, 1.3, and 0.342 kDa).

Additionally, monosaccharide compositions of EPS-LB13 and EPS-MLB10 were determined after hydrolyzing the purified EPS using 2 M trifluoroacetic acid (TFA) at 105 °C for 2 h in a heating block, followed by 1-phenyl-3-methyl-5-pyrazolone (PMP) derivatization, according to (Vojvodić Cebin, Komes, & Ralet, 2022). Briefly, the derivatized EPS was analyzed by injecting the filtered samples through Thermo C18 (250 × 4.6 mm, 5 μm) column in Shimadzu HPLC system equipped with an SPD-M20A photodiode array detector (PDA-245 nm). A gradient program with varied mobile phase (A: 0.1 M Ammonium acetate, Mobile Phase B: Acetonitrile) concentration at flow rate: 1.5 mL/min and column temperature of 30 °C was used.

#### 2.3.2 Ultraviolet (UV) and Fourier transform-infrared spectroscopy (FT-IR)

The Fourier transform-infrared spectroscopy (FT-IR) analysis of the purified EPS-LB13 and EPS-MLB10 was carried out to determine the functional groups within the EPS structures using previously described methodology (Ali et al., 2023). Briefly, the EPS was placed on a Diamond/ZnSe crystal plate of Spectrum Two FT-IR Spectrometer (PerkinElmer Inc., USA) and FT-IR spectra was obtained by scanning for16 times from 4000 to 400 cm^−1^ at room temperature (23 ± 0.1 °C) with a scanning resolution of ± 4 cm^−1^. To detect the purity of EPS-LB13 and EPS-MLB10, both EPSs were diluted to 1 mg/mL using deionized water in a quartz cuvette (Hellma, Germany) and purity was checked by ultraviolet spectral scanning at 200–800 nm using BioTek Epoch 2 Microplate Spectrophotometer (Agilent Technologies, Inc., USA) (Zhu et al., 2019).

#### 2.3.3 Zeta potential and particle size analysis

The zeta potential and the particle size of the EPS were analyzed according to (Ayyash et al., 2020). For this, the purified EPS-LB13 and EPS_MLB3 were diluted to 1 mg/mL using deionized water and placed in the cells of NanoPlus-3 Particulate Systems for the analysis at 25 °C (Micromeritics Instrument Corp., USA).

### 2.4 Evaluation of bioactive characteristics of EPS

#### 2.4.1 Antioxidant capacity

##### 2.4.1.1 Radical scavenging by DPPH and ABTS analysis

The radical scavenging activities of purified EPSs were evaluated according to (Sharma, 2015) using 1,1-diphenyl-2-picrylhydrazyl (DPPH) and 2,2′-azino-bis(3-ethylbenzene-thiazoline-6-sulphonic acid) radical (ABTS•+) tests, and the scavenging rates were calculated using the following equation:

$$\mathrm{Scavengingrate}\%=\frac{\left(Absorbanceblank-AbsorbanceEPS\right)}{\mathrm{Absorbanceblank}}\times 100$$

##### 2.4.1.2 Superoxide scavenging activity

The superoxide scavenging activities of EPS-LB13 and EPS-MLB10 were determined by investigating superoxide dismutase (SD) and superoxide anion scavenging (SAS) activities, which were measured according to the method described by (Siswoyo, Arum, Sanjaya, & Aisyah, 2021) at 420 nm and 320 nm absorbance, respectively. The rate of inhibition of pyrogallol oxidation was calculated by:

$$\mathrm{Scavengingrate}\%=\frac{\left(Absorbanceblank-AbsorbanceEPS\right)}{\mathrm{Absorbanceblank}}\times 100$$

##### 2.4.1.3 Reactive oxygen scavenging activity

Hydrogen peroxide and hydroxyl radical scavenging activity tests of the purified EPS-LB13 and EPS-ML10 were performed as described by (Wang et al., 2012) to determine the inhibition of the reactive oxygen at 230 nm and 536 nm, respectively,. The scavenging activity was expressed as a percentage using the equation:

$$\mathrm{Scavengingrate}\%=\frac{\left(Absorbanceblank-AbsorbanceEPS\right)}{\mathrm{Absorbanceblank}}\times 100$$

##### 2.4.1.4 Metal chelation

The ability of purified EPS-LB13 and EPS-MLB10 to prevent the oxidative stress was determined by metal chelating activity and inhibition of lipid peroxidation tests according to (Sirin & Aslim, 2020). The inhibition of the oxidative stress was calculated using the scavenging rate equation:

$$\mathrm{Scavengingrate}\%=\frac{\left(Absorbanceblank-AbsorbanceEPS\right)}{\mathrm{Absorbanceblank}}\times 100$$

##### 2.4.1.5 Antioxidant power analysis

The antioxidant power analysis of EPS-LB13 and EPS-MLB10 was carried out using ferric reducing antioxidant power (FRAP) and reducing power (RP) tests as described previously (Sharma, 2015). The standard graphs of ascorbic acid (100–1000 µg/mL) were plotted at 593 nm and 700 nm for FRAP and RP, respectively. The results were calculated in terms of µg/mL equivalent to ascorbic acid.

##### 2.4.1.6 Total antioxidant analysis (TAC)

The Total Antioxidant Capacity (TAC) was measured according to (Adebayo-Tayo, Ishola, & Oyewunmi, 2018). Briefly, an aqueous TAC solution (4 mM ammonium molybdate, 600 mM sulfuric acid, and 28 mM sodium sulfate) was mixed with theEPS-LB13 and EPS-MLB3. The absorbance was measured at 695 nm after 15 min of incubation at room temperature. A standard graph was plotted for ascorbic acid (50–1000 µg/mL), and the antioxidant capacity was calculated using the slope equation for EPS in µg/mL equivalent to ascorbic acid.

#### 2.4.2 Minimum inhibitory concentrations and antibiofilm activity tests of EPSs

The inhibitory activity of EPS-LB13 and EPS-MLB3 against *Escherichia coli* 0157:H7 1934*, S.* Typhimurium 02–8423*, Staphylococcus aureus*, and *Listeria monocytogenes* DSM 20649 and antibiofilm activity tests were performed using previously described methodologies (Tarique et al., 2022, Wang et al., 2015). Biofilm inhibition was measured at 590 nm using the equation:

$$\mathrm{AntibiofilmActivity}\%=[1-\left(\frac{\mathrm{AbsorbanceEPS}}{\mathrm{Absorbanceblank}}\right)]\times 100$$

#### 2.4.3 Cytotoxic activity test of EPSs

Cytotoxic effects of purified EPS-LB13 and EPS-MLB10 on the Caco-2 and MCF-7 cancer cell lines were evaluated using the method described by (Ayyash, Al-Nuaimi, Al-Mahadin, & Liu, 2018). Cytotoxicity was calculated using the following formula:

$$\mathrm{Cytotoxicity}\%=100-\left(\frac{\mathrm{AbsorbanceEPS}-AbsorbanceBlank}{\mathrm{AbsorbanceControl}-AbsorbanceBlank}\right)\times 100$$

#### 2.4.4 Inhibition of α-amylase and α-glucosidase activities

The inhibition of α-amylase and α-glucosidase was used to assess the antidiabetic activity of purified EPS-LB13 and EPS-MLB10 according to (Ayyash et al., 2018). The absorbance was recorded at 540 and 400 nm for α-amylase and α-glucosidase, respectively. The rate of inhibition was calculated using the following equation:

$$\mathrm{Inhibiton}\%=\left(\frac{\mathrm{Absorbanceblank}-AbsorbanceEPS}{\mathrm{Absorbanceblank}}\right)\times 100$$

#### 2.4.5 Angiotensin-converting enzyme (ACE) inhibition

Purified EPS-LB13 and EPS-MLB10 were evaluated for ACE inhibitory activity according to (Ayyash et al., 2018). The inhibition was calculated using the following equation:

$$\mathrm{ACEInhibition}\%=\left(\frac{\mathrm{AbsorbanceBlank}-AbsorbanceEPS}{\mathrm{AbsorbanceBlank}}\right)\times 100$$

#### 2.4.6 Cholesterol-lowering Activity

To determine the cholesterol removal activity of EPS-LB13 and EPS-MLB10, the method described by (Yılmaz & Şimşek, 2020) was used, and absorbance was recorded at 500 nm. The rate of cholesterol lowered was calculated using the following formula:

$$\mathrm{Cholesterollowered}\%=\left(\frac{\mathrm{AbsorbanceBlank}-AbsorbanceEPS}{\mathrm{AbsorbanceBlank}}\right)\times 100$$

### 2.5 Prebiotic properties of EPS-LB13 and EPS-MLB10

The prebiotic effects of the purified EPS-LB13 and EPS-MLB10 on 12 probiotic strains; *Lactobacillus acidophilus* DSMZ 9126, *Bifidobacterium longum* subsp. *longum* DSMZ 20079, *Lactobacillus delbrueckii* subsp. *delbrueckii* DSMZ 20074, *Lactobacillus delbrueckii* subsp. *lactis* DSMZ 20076, *Lactobacillus rhamnosus* DSMZ 20021, *Lactobacillus paracasei* subsp. *paracasei* DSMZ 20207, *Lactobacillus plantarum* DSMZ 2648, *Lactobacillus paracasei* subsp. *tolerans* DSMZ 20258, *Lactobacillus gasseri* DSMZ 20243, *Lactobacillus delbrueckii* subsp. *bulgaricus* DSMZ 20081, *Bifidobacterium breve* DSMZ 20213, and *Bifidobacterium animalis* subsp. *lactis* DSMZ 10140 were determined using previously described methodology (Yılmaz & Şimşek, 2020). The growth kinetics of each probiotic strain with different carbon sources individually were measured at 600 nm for 24 h at 15 min intervals.

### 2.6 In vitro fecal fermentation

#### 2.6.1 In vitro fecal fermentation of EPS-LB13 and EPS-MLB10

Fecal slurry was prepared from fresh fecal samples collected from healthy individuals aged 24–40 who had not recently been administered antibiotics. Fecal fermentation was performed following the method of (Yi et al., 2022) by mixing fecal slurry with basal medium and different treatments (blank with no additional carbon source as negative control, galacto-oligosaccharides (GOS-P) as a positive control, purified EPS-LB13, and EPS-MLB10) and incubating them at 37 °C in a shaking water bath for 24 h.

#### 2.6.2 Determination of broth properties during fecal fermentation

The changes in pH, total sugar, gas production, and reducing sugar levels during fecal fermentation of purified EPS-LB13 and EPS-MLB10 were examined according to (Ding et al., 2019) at 0, 6, 12 and 24 h of fermentation period.

#### 2.6.3 Microbial analysis during fecal fermentation

The microbial composition of each group (Blank, GOS-P, EPS-LB13, and EPS-ML10) was analyzed at each time point (0, 6, 12, and 24 h) during fecal fermentation. Genomic DNA was extracted using a Genomic DNA Kit (Tiangen, Beijing, China) and V3-V4 regions of 16SrRNA were amplified and analyzed by BGI, Hong Kong. Library construction, concentration, and quality assessment were performed using Agencourt AMPure XP beads and Agilent 2100 Bioanalyzer, respectively. The raw data were filtered using iTools Fqtools fqcheck (v.0.25), and the paired-end reads were merged into a single tag sequence using Fast Length Adjustment of SHort reads (FLASH, v1.2.11) (Magoč & Salzberg, 2011). The sequences were clustered into operational taxonomic units (OTUs) with 97 % similarity threshold by UPARSE, and chimeras were removed using UCHIME (v4.2.40). The OTU representative sequences were mapped to the tags using USEARCH (v7.0.1090) and aligned against the database for taxonomic annotation using the RDP classifier (v2.2) at 60 % sequence identity. The alpha diversity was calculated using mothur (v.1.31.2), beta diversity was obtained using QIIME (v1.80) and R (v3.1.1), differential species analysis was carried out using Linear discriminant analysis Effect Size (LEfSe) (https://huttenhower.sph.harvard.edu/galaxy/), microbial functional annotation was predicted by PICRUSt2 v2.3.0-b, and R(v3.4.10), and correlation analysis and model prediction were performed using R(v3.4.1) and Cytoscape.

#### 2.6.4 Short-chain fatty acids (SCFAs) production during fecal fermentation

The production of SCFAs during the fecal fermentation of purified EPS-LB13 and EPS-MLB10 was measured according to (Dobrowolska-Iwanek et al., 2020) with some modifications. After 24 h of fecal fermentation, the broth was centrifuged at 15000×g for 20 min, and the supernatant was filtered using 0.45 µm filters. The Shimadzu HPLC system equipped with an SPD-M20A photodiode array detector (PDA) was used for SCFAs analysis. For this, a Shodex C18M 4E (250 × 4.6 mm, 5 μm) column (Resonac Inc, Japan) was utilized and column conditions were an isocratic mobile phase containing 10 mM Monopotassium Phosphate, pH 2.4 with phosphoric acid and 100 % Acetonitrile (80:20) at a flow rate of 1.5 mL/min and a column temperature of 30 °C. The injection volume was 20 μL and the run time was set to 7 min with the UV detector set at 210 nm. Standard curves were prepared for acetic acid, propionic acid, and butyric acid under similar conditions at different concentrations.

### 2.7 Rheological properties of fermented bovine milk

The rheological properties of skimmed bovine milk fermented with *E. faecium* (EPS-LB13) and *S. thermophilus* (EPS-MLB10) were measured in a Pelt AR2000 concentric cylinder with a bob apparatus using a Discovery HR-2 Hybrid rheometer (TA Instruments, USA). Sterilized skimmed bovine milk was transferred to the cup geometry, and the gap was set to 100 µm. The samples were measured at a constant temperature of 44 °C for 14,400.0 s, with a sampling interval of 60.0 s/pt and a strain of 0.1 %. The analysis was conducted at a single-point frequency of 0.5 Hz. The samples were then conditioned for 45 min at 5 °C to allow yogurt to set. After 45 min, a frequency sweep test was performed at 5 °C to evaluate the viscoelastic behavior of the samples. The frequency varied from 0.1 to 100 Hz at a strain of 0.5 % within the linear viscoelastic region. The points per decade were set at 30. The viscoelastic behavior of the fermented milk samples was evaluated using storage modulus (G′) and loss modulus (G″). Data were analyzed using TRIOS 5.2 software. Fermentation was performed for the Starter culture-Yo-Flex Chr. Hansen culture (SC), SC + *E. faecium* (EPS-LB13), and SC + *S. thermophilus* (EPS-MLB10).

### 2.8 Statistical analysis

All experiments were performed in triplicates. The effects of purified EPS extracted from *Enterococcus faecium* MW725386 and *Streptococcus thermophilus* MW725391 on the activities mentioned above were analyzed by conducting a statistical analysis of the differences among the concentrations (100, 150, 200, and 250 µg/L). One-way ANOVA was performed using Minitab 19.0 (Minitab LLC, PA, USA), and Tukey's test was used to compare between means with a significance level of *P < 0.05*.

## 3 Results and discussion

### 3.1 Purification of EPS-LB13 and EPS-MLB10

EPS-LB13 and EPS-MLB10 were extracted from culture supernatants of *E. faecium* MW725386 and *S. thermophilus* MW725391, respectively, as Labaneh isolates (Tarique et al., 2022). UV-spectral scanning of EPS-LB13 and EPS-MLB10 showed no peaks between 260 and 290 nm, indicating the absence of proteins and nucleic acids, and a single peak at 220 nm was observed as a typical peak for EPS (data not shown). The purified EPSs were water-soluble and had a yield of 257.32 ± 31.65 mg/L (EPS-LB13) and 271.53 ± 20.10 mg/L (EPS-MLB10), respectively, as calculated by the phenol–sulfuric acid method (Dubois et al., 1956). These values are comparable to those reported in other studies on EPS-producing LAB strains (Bhat and Bajaj, 2018, Jia et al., 2019).

### 3.2 Characterization of EPS-LB13 and EPS-MLB10

The molecular weight and monosaccharide composition of purified EPS-LB13 and EPS-MLB10 were analyzed by GPC and HPLC analysis, respectively. The results indicated that the purified EPS-LB13 and EPS-MLB10 had molecular weights of 1975 and 1553 kDa, respectively, as determined using the standard curve (Fig. S1a) of Pullulan standards. These levels of EPS molecular weight were also reported in previous observations, although lower and higher EPS molecular weights were also reported (Ayyash et al., 2020, Jia et al., 2019, Leivers et al., 2011, Yilmaz et al., 2022). Exploring distinct EPSs with different levels of molecular weight could be interesting for the usage of these EPSs for distinct applications such as production of food products with varied viscous behaviors. Furthermore, both EPS samples had different monosaccharide compositions, as determined by comparing their chromatograms with the PMP-derivatized standard monosaccharide retention time (Fig. S1b). EPS-LB13 (Fig. S1c) contained galacturonic acid, galactose, glucose, mannose, and xylose in a molar ratio of 13:45:61:26:1, whereas EPS-MLB10 (Fig. S1d) contained glucose, ribose, mannose, and xylose in a molar ratio of 8:13:6:1. These results suggest that different LABs can produce different EPS with varying molecular weights and monosaccharide compositions. Previous studies also reported the presence of different sugar monomers in EPS repeating unit structures of distinct *E. faecium* (Ayyash et al., 2020, Jia et al., 2019) and *S. thermophilus* strains, suggesting the role of strain-specific conditions for the final EPS structures (Al-Nabulsi et al., 2022, Li and Shah, 2016).

The FT-IR spectra of the EPS-LB13 and EPS-MLB10 are shown in Fig. S2. The spectra revealed the presence of typical polysaccharide functional groups, such as hydroxyl, C—H stretching, vibration of aliphatic CH_2_ and glycosidic linkages. The main differences between the two EPS samples were observed in the pronounced peaks at approximately 579 cm^−1^, 882 cm^−1^, 812 cm^−1^, 675 cm^−1^ and 507 cm^−1^, which indicated the presence of different functional groups or monosaccharides in the EPS structure. The peak at approximately 882 cm^−1^ for EPS-MLB10 was due to the C—O—C bending vibration of the pyranose ring, which may indicate the presence of xylose in its structure. The peak around 579 cm^−1^ for EPS-LB13 was due to the C—O bending vibration of carboxyl or ester groups, which may indicate the presence of galactose in its structure. Both these differences can be related to the monosaccharide composition (Hu et al., 2021). The FT-IR spectra of EPS-LB13 and EPS-MLB10 were slightly different from those of EPS obtained from other *S. thermophilus* (Al-Nabulsi et al., 2022) and *E. faecium* strains (Ayyash et al., 2020), supporting the role of strain specific conditions for the final EPS structures. Moreover, the EPS-LB13 had a smaller particle size (65.3 nm) and a lower zeta potential (−1270.97 mV) than EPS-MLB10 (35.2 nm and − 25.11 mV, respectively). These results might suggest that EPS-LB13 has a higher degree of ionization and hydration than EPS-MLB10, which may be related to their different monosaccharide compositions and molecular weights. The zeta potential and particle size of EPSs may affect their stability, solubility, and interaction with other molecules in the solution. Overall, the physicochemical characterization of both EPSs demonstrated their varying molecular weights, monosaccharide compositions, functional groups, zeta potentials and particle sizes. These properties may influence the biological activities and functional properties of EPSs (Abdalla et al., 2021, Tiwari et al., 2021, Zhu et al., 2019).

### 3.3 Bioactive characteristics of EPS-LB13 and EPS-MLB10

The bioactive roles of the purified EPSs were tested in terms of antioxidant, anticancer, antidiabetic, antimicrobial, cholesterol, and ACE inhibition activities. The antioxidant effects of purified EPS-LB13 and EPS-MLB10 were assessed using various methods to measure their abilities to scavenge different types of free radicals, chelate metal ions, reduce oxidizing agents, and prevent lipid peroxidation (Table 1). Previously, the *in vitro* antioxidant evaluation of EPSs demonstrated its ability to protect cells from oxidative damage caused by free radicals, metal ions, oxidizing agents, and lipid peroxidation, which are involved in various pathological processes such as inflammation, ageing, and cancer (Andrew & Jayaraman, 2020). The radical scavenging activities of EPS-LB13 and EPS-MLB10 were 26 % and 41 %, respectively, for DPPH, and 41 % and 36 %, respectively, for ABTS at a concentration of 250 mg/L of EPS. These findings demonstrated the promising antioxidant nature of both EPSs as these levels of antioxidant capacity were reported for higher concentrations of EPSs from other LAB isolates (Jia et al., 2019, Lobo et al., 2019). Purified EPSs has also been used to investigate the effect of superoxide radicals, which are precursors for the production of hydrogen peroxide and oxygen, which may disrupt oxidative homeostasis (Zhang et al., 2016). The superoxide dismutase activities of EPS-LB13 and EPS-MLB10 at 250 mg/L was 45 % and 54 %, respectively, while the superoxide anion scavenging activity was 56 % and 62 %, respectively, which were comparable to those reported in previous studies (Jia et al., 2019, Xu et al., 2023). The reactive oxygen scavenging (ROS) activities of purified EPS were measured using hydrogen peroxide and hydroxyl radical scavenging activities. The hydrogen peroxide scavenging activity was 10 % and 11 % for EPS-LB13 and EPS-MLB10, respectively, whereas the hydroxyl radical scavenging activities were 86 % and 82 %, respectively, at 250 mg/L EPS, which were higher than those of the EPS isolated from *S. thermophilus* GST-6 (Xu et al., 2023) and *E. faecalis* (Choudhuri et al., 2020). Our results are comparable to antioxidant activities reported by Wang et al. (2023), who characterized heteropolysaccharides from *Pteridium revolutum*. The ability of purified EPS to inhibit oxidative stress caused by the chelation of metal ions and lipid peroxidation was also measured. EPS-LB13 and EPS-MLB10 at 250 mg/L inhibited lipid peroxidation by 59 % and 67 %, respectively, and metal chelation by 75 % and 85 %, respectively, which were similar to those of *Enterococcus faecalis* (Choudhuri et al., 2020). The antioxidant powers of purified EPS-LB13 and EPS-MLB10 was expressed as µg/mL equivalent to that of ascorbic acid, which is a well-known antioxidant. The FRAP, TAC, and RP values of EPS-LB13 and EPS-MLB10 at 250 mg/L were 1219 µg/mL, 2592 µg/mL and 714 µg/mL, respectively, and 1553 µg/mL, 2848 µg/mL and 792 µg/mL, respectively. The antioxidant power and total antioxidant values were comparable to those reported in previous studies of EPS extracted from various *Enterococcus* and *Streptococcus* species (Choudhuri et al., 2020, Lobo et al., 2019). Based on the results, all antioxidant properties were directly proportional to the concentration, as the antioxidant properties increased a dose-dependent manner (*P < 0.05*). EPS-MLB10 had a stronger antioxidant activity than EPS-LB13 in most assays which might be related to the alteration of the physiochemical properties of these EPSs such as molecular weight, net negative charge, and presence of certain functional groups and monosaccharides. The strong antioxidant capacity of both EPSs might be important for their potential use in the food and health industries as discussed previously (Andrew and Jayaraman, 2020, Tiwari et al., 2021, Zhu et al., 2019).

**Table 1:** In vitro Antioxidant Activities of EPS-LB13^1^ and EPS-MLB10^2^ at different concentrations (mg/L).

	DPPH (%)	ABTS (%)	SD (%)	SAS (%)	HP (%)	HRS (%)	MC (%)	LO (%)	FRAP (µg/mL)	TAC(µg/mL)	RP (µg/mL)
EPS-LB13 (mg/L)^1^											
100	21.9 ± 0.78^c^	34.2 ± 1.30^c^	19.4 ± 0.50^d^	40.9 ± 0.21^d^	8.6 ± 0.10^c^	6.4 ± 0.90^d^	35.6 ± 1.45^d^	8.3 ± 0.85^d^	169.5 ± 13.75^d^	632.9 ± 12.86^d^	4.76 ± 1.65^d^
150	22.7 ± 0.68^b^	36.1 ± 0.26^b^	29.1 ± 1.41^c^	44.3 ± 0.26^c^	8.9 ± 0.04^c^	48.1 ± 0.43^c^	45.9 ± 0.84^c^	12.8 ± 0.24^c^	307.0 ± 19.53^c^	1104.2 ± 14.00^c^	274.29 ± 6.55^c^
200	24.9 ± 0.68^a^	36.2 ± 1.12^b^	35.2 ± 0.62^b^	52.9 ± 0.20^b^	9.0 ± 0.04^b^	79.5 ± 0.43^b^	68.2 ± 0.66^b^	34.1 ± 0.85^b^	637.0 ± 15.46^b^	1601.5 ± 14.74^b^	454.76 ± 6.75^b^
250	25.9 ± 1.40^a^	41.0 ± 1.24^a^	45.0 ± 0.35^a^	56.4 ± 0.13^a^	10.3 ± 0.12^a^	85.6 ± 0.30^a^	74.8 ± 1.26^a^	58.8 ± 1.54^a^	1219.0 ± 10.29^a^	2592.2 ± 14.00^a^	714.29 ± 5.71^a^
EPS-MLB10 (mg/L)^2^											
100	23.9 ± 1.04^d^	16.6 ± 1.12^d^	37.4 ± 1.06^c^	58.2 ± 0.16^b^	9.7 ± 0.09^c^	5.60 ± 1.03^d^	66.2 ± 1.52^d^	46.4 ± 0.72^d^	514.5 ± 7.28^d^	838.2 ± 9.17^d^	109.05 ± 5.02^d^
150	27.4 ± 0.78^c^	22.0 ± 0.90^c^	45.7 ± 0.89^b^	61.4 ± 0.14^a^	10.2 ± 0.09^b^	49.0 ± 0.39^c^	69.0 ± 0.77^c^	55.2 ± 1.42^c^	770.6 ± 8.67^c^	1329.5 ± 7.57^c^	244.76 ± 5.95^c^
200	38.7 ± 0.57^b^	31.3 ± 0.79^b^	51.8 ± 0.64^a^	61.9 ± 0.19^a^	10.5 ± 0.03^b^	78.0 ± 0.28^b^	73.4 ± 0.75^b^	59.2 ± 1.09^b^	1179.8 ± 34.41^b^	1842.9 ± 5.01^b^	462.38 ± 7.05^b^
250	41.0 ± 0.31^a^	36.5 ± 0.79^a^	54.3 ± 0.64^a^	62.3 ± 0.03^a^	11.1 ± 0.08^a^	81.8 ± 0.73^a^	85.1 ± 1.16^a^	66.9 ± 1.19^a^	1552.9 ± 7.28^a^	2848.2 ± 7.21^a^	792.38 ± 5.95^a^

^(a–d)^ Means of the values in the same column, but with different superscripts, are significantly different from each other (*P < 0.05*).

1 EPS produced by *Enterococcus faecium* MW725386.

2 EPS produced by *Streptococcus thermophilus* MW725391.

**Fig. 1:**
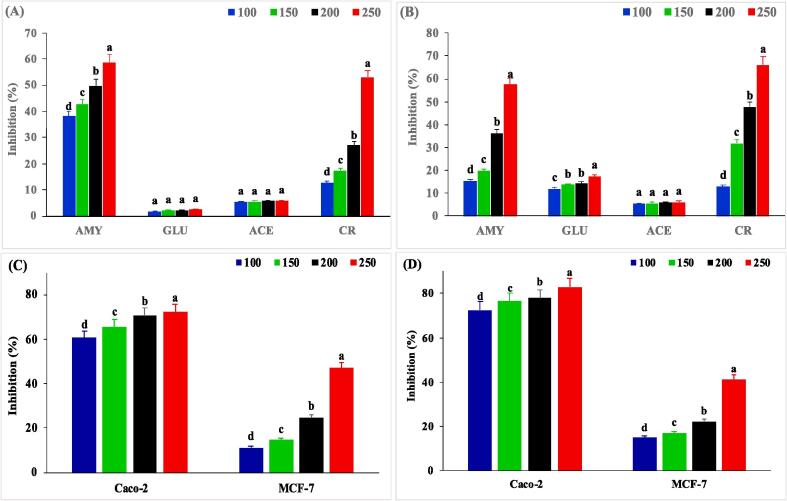
In vitro Bioactivities of EPS-LB13 and EPS-MLB10 at different concentrations (mg/L). AMY: α-Amylase, GLU: α-Glucosidase, ACE: Angiotensin-Converting Enzyme, and CR: Cholesterol Removal of (A) EPS-LB13, (B) EPS-MLB10. Anticancer Activities on Caco-2 and MCF-7 Cell Lines of EPS-LB13 (C) and EPS-MLB10 (D). Bars are means ± standard deviations (error bars). ^a-d^ Means with different lowercase letters at the same parameter differed significantly (*P < 0.05*).

The effects of purified EPS-LB13 and EPS-MLB10 on α-amylase, α-glucosidase, ACE, and cholesterol levels are shown in Fig. 1a and Fig. 1b, respectively. The inhibition of both diabetic-causing enzymes increased (*P < 0.05*) with increasing concentrations of EPS, indicating potential antidiabetic properties by inhibiting the key enzymes involved in carbohydrate digestion and absorption (Ali et al., 2023). The strongest inhibition was observed at 250 mg/L, where EPS-LB13 inhibited α-amylase and α-glucosidase by 59 % and 2 %, respectively, while EPS-MLB10 inhibited them by 57 % and 17 %, respectively. A possible reason for the lower inhibition of α-glucosidase compared to the previous studies by the same species (Al-Nabulsi et al., 2022, Ayyash et al., 2020) may be the concentration of EPS, and the trend of increasing inhibition was consistent. Similar trends were observed in the inhibition of cholesterol levels and the role of EPSs were also concentration-dependent (*P < 0.05*). Cholesterol inhibition at 250 mg/L for EPS-LB13 and EPS-MLB10 was 53 % and 66 %, respectively, and the inhibitions of ACE were relatively low and did not show a clear trend with increasing concentrations of EPS for both strains. The inhibition of ACE was around 6 % at 250 mg/L, indicating that EPS may not have significant antihypertensive properties, contrary to the EPS from *S. thermophilus* and *L. bulgaricus* which had around 78 % of ACE inhibition (Al-Nabulsi et al., 2022). The difference in the inhibition of α-amylase, α-glucosidase, cholesterol and ACE by the same EPS may be due to the different substrate specificities and mechanisms of action of the enzymes (Ayyash et al., 2020).

The cytotoxic activities of EPSs on Caco-2 and MCF-7 cancer cell lines are shown in Fig. 1c and Fig. 1d, where a continued trend of increasing (*P < 0.05*) cytotoxicity of both EPS on the cell lines was observed, which indicated potential anticancer properties of EPSs by inducing cell death or inhibiting cell proliferation (Andrew & Jayaraman, 2020). The cytotoxicity of EPS-LB13 at 250 mg/L on Caco-2 and MCF-7 cells was 81 % and 83 %, respectively, which was similar to that of EPS obtained from *S.therompillus* (Li & Shah, 2016). In contrast, EPS-MLB10 demonstrated lower cytotoxicity on MCF-7 cells (14 %) than on Caco-2 cells (83 %) which was comparable to the EPS produced by *E. faecalis* (Ali et al., 2023). The antimicrobial and antibiofilm activities of EPS-LB13 and EPS-MLB10 against common food pathogens were evaluated at three different concentrations (250, 125, and 62.5 mg/L). The results are shown in Fig. S3a and Fig. S3b. The MIC values are defined as the lowest concentration of EPS that inhibited at least 50 % of the visible growth of the pathogens after 24 h of incubation at 37 °C (Tarique et al., 2022). The antimicrobial activities of EPS-LB13 and EPS-MLB10 at 250 mg/L against various food pathogens were *E. coli* 0157:H7 (63 % and 57 %), *S.* Typhimurium (63 % and 74 %), *S. aureus* (53 % and 62 %), and *L. monocytogenes* DSM 20649 (55 % and 62 %), respectively. Fig. S3c and Fig. S3d, show the antibiofilm activities of EPS-LB13 and EPS-MLB10 at 250 mg/L against the same foodborne pathogens were *E. coli* 0157:H7 (55 % and 59 %), *S.* Typhimurium (55 % and 61 %), *S. aureus* (55 % and 57 %), and *L. monocytogenes* (54 % and 58 %), respectively. The results were similar to those of *Enterococcus* strains from previous studies (Bhat & Bajaj, 2018). The bioactive and functional properties of the EPSs, including antioxidant, antidiabetic, anticancer, antimicrobials, and others, are highly dependent on the characteristics and sources of the EPS (Abdalla et al., 2021, Andrew and Jayaraman, 2020). Therefore, EPS-LB13 and EPS-MLB10 could be considered promising carbohydrate polymers with multiple bioactive and functional roles for potential applications in the food and health industries.

### 3.4 Prebiotics functions of EPS-LB13 and EPS-MLB10

To test the prebiotic effects of EPS-LB13 and EPS-MLB10, first, the growth-promoting functions of both EPSs on 12 probiotic strains were measured by determination of their growth kinetics in the presence of EPSs as carbon sources. The important criteria for any compound to be considered as a prebiotic are digestibility by gut microbes and the improvement of the host’s health (Tiwari et al., 2021). As seen in Fig. S4, it was clear that the pure cultures of the various probiotics utilized EPS as compared to glucose, showing their prebiotic activities. The max V values were similar to those of glucose and EPS, but the lag time and the time to reach maximum growth decreased for all the probiotics, which indicates that the EPS reduced the time to adapt to the carbon source and start growing exponentially. However, EPS-MLB10 had longer lag time than glucose for *L. rhamnosus*, *L. paracasei* subsp. *tolerans*, and *L. gasseri*, with max V slightly lower than those of glucose. These results suggest that EPS-LB13 was better than EPS-MLB10 when replaced as a carbon source for probiotic growth determination. The results were comparable to those of a previous study on EPS (Yılmaz & Şimşek, 2020), and the differences in the EPS prebiotic activity may be due to the characteristics, specifically the easy degradation by probiotics due to their structural conformation (Bello et al., 2001).

### 3.5 Fecal fermentation properties of EPS-LB13 and EPS-MLB10

#### 3.5.1 Effect of EPS-LB13 and EPS-MLB10 on fecal fermentation broth

**Fig. 2:**
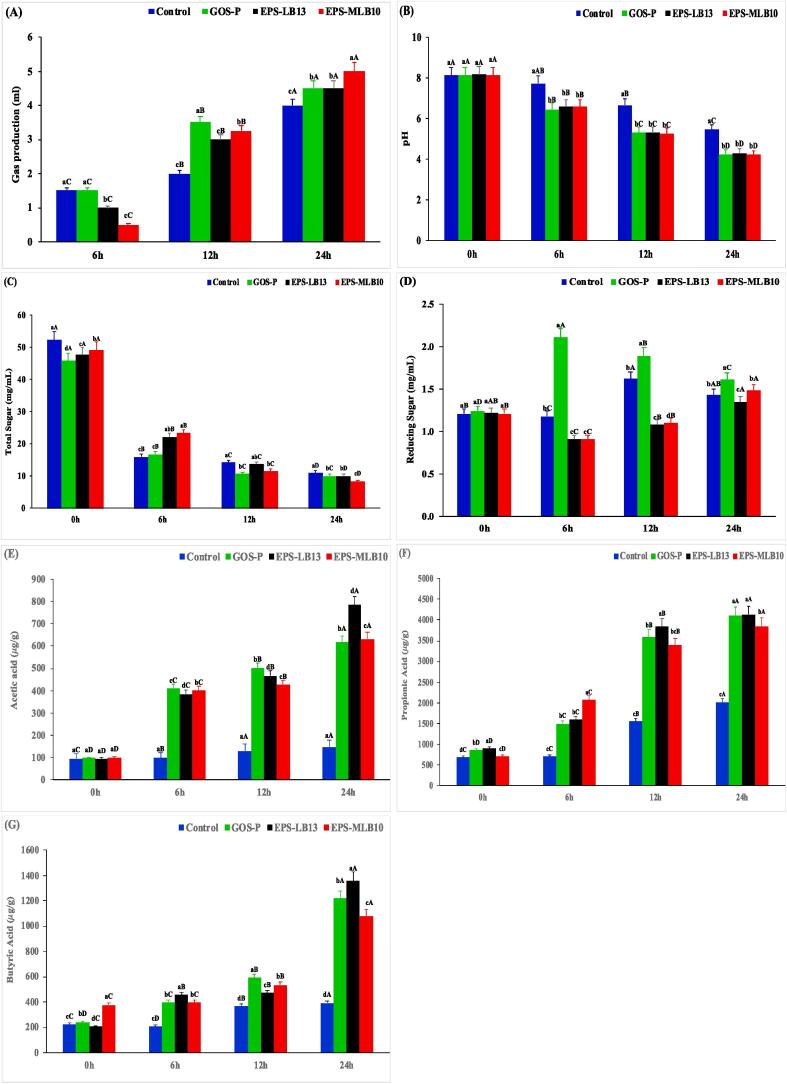
Effect of EPS during 0, 6, 12, and 24 h of Fecal Fermentation for Gas Production (A), pH (B), Total Sugar (C), Reducing Sugar (D), Acetic Acid Production (E), Propionic Acid Production (F), and Butyric Acid Production (G) of the Sample Groups: Blank (negative control), GOS-P (positive control), EPS-LB13, and EPS-MLB10. Bars are means ± standard Deviations (Error Bars).^a–d^ Means with different lowercase letters at the same time differed significantly (*P < 0.05*). ^A–D^ Means with different uppercase letters, at different times and same treatment, differed significantly (*P < 0.05*).

The effect of the purified EPS-LB13 and EPS-MLB10 on *in vitro* fecal fermentation broth was evaluated for gas production, pH changes, reducing sugars, total sugars, and SCFA production (Fig. 2a-g). The known prebiotic galacto-oligosaccharide (GOS-P) and the blank without addition of carbohydrates were used as a positive and negative controls, respectively. The gas production (Fig. 2a) after 6 h of fecal fermentation in the blank, GOS-P, EPS-LB13 and EPS-MLB10 groups were 1.5, 1.5, 1 and 0.5 mL, respectively, that increased to 4, 4.5, 4.5 and 5 mL, respectively, after 24 h and no significant difference was observed among the groups. The production of gas was observed due to gut bacteria that break down undigested carbohydrates and other food components, and these gases can build up in the intestines, causing digestive discomfort (Dobrowolska-Iwanek et al., 2020). The pH values (Fig. 2b) were similar to that for gas production, with no significant (*P < 0.05*) difference after 6 h of fecal fermentation, except for the blank, which had the highest pH value (7.7). After 24 h, a sharp decrease in the pH value was observed in the blank, GOS-P, EPS-LB13, and EPS-MLB10 groups that were 5.4, 4.2, 4.3, and 4.2, respectively, which indicated the breakdown of sugars to acids. The total sugars (Fig. 2c) after 24 h of incubation were lowest in the EPS-MLB10 group and highest in the blank group, 8.3 and 11 mg/mL. This trend may be attributed to the consumption of these sugars by gut microbiota during fecal fermentation (Fu et al., 2018, Xie et al., 2019). Fig. 2d demonstrates that the amount of reducing sugars in EPS-LB13 and EPS-MLB10 decreased significantly (*P < 0.05*) after 6 h and 14 h of fecal fermentation, and then slightly increased after 24 h. However, the reducing sugar content in GOS-P samples increased after 6 h of fermentation, followed by a decrease (*P < 0.05*) after 12 h and 24 h of fermentation. Similarly, Wu et al. (2022) reported an increase in reducing sugar during the 24 h followed by a decrease after 48 h of fermentation. This contradicts with other reports that reducing sugars decreased after 12 h of fecal fermentation. We propose that the complexity of the current EPSs might retard its degradation by the gut microbiota during 24 h of fecal fermentation. The pH decreased, total sugar decreased, and gas increased, indicating gut microbiota consumed indigestible EPSs during fecal fermentation (Wu et al., 2022).

The SCFA production in fecal fermentation may be due to the digestion of carbohydrates by the gut microbiome, which is evident from the production of gas and decrease in the pH and sugar contents, as shown in (Fig. 2a-d). Acetic acid, propionic acid and butyric acid are the main SCFAs produced during fecal fermentation, which are known to provide energy to the colon, protect the lining of the gut and regulate the immune system, respectively (Fu et al., 2018). The SCFAs production during fermentation is shown in Fig. 2e-g. The total SCFAs production after 24 h of fermentation was similar (*P < 0.05*) among the groups GOS-P, EPS-LB13 and EPS-MLB10, with values ranging from 5548 to 6023 PPM. The blank had the lowest (*P < 0.05*) total SCFA production (4530 PPM), as expected due to the lack of carbohydrate addition. The acetic acid production Fig. 2e, was the least in all the groups compared to propionic acid and butyric acid during fermentation. The acetic contents increased (*P < 0.05*) during fecal fermentation time in GOS-P, EPS-LB13, and EPS-MLB10 and reached up to 616.2, 784.5, and 630.6 (μg/g), respectively. After 24 h, EPS-LB13 group had the highest (*P < 0.05*) acetic acid content (Fig. 2e). The propionic acid production Fig. 2f, was higher in all the groups than acetic acid and butyric acid during fermentation, with EPS-LB13 having the highest (P < 0 0.05) propionic acid production (4192 μg/g) after 24 h of fermentation, followed by blank (4138 μg/g), EPS-MLB10 (3858 μg/g) and GOS-P (3853 μg/g). The butyric acid production (Fig. 2g) was higher in all the groups than acetic acid during fermentation, but lower than propionic acid, except for EPS-MLB10 which had the highest (*P < 0.05*) butyric acid production (1534 μg/g) after 24 h of fermentation, followed by GOS-P (1078 μg/g), EPS-LB13 (1041 μg/g) and blank (392 μg/g). Acetic acid has shown promise in enhancing the body's glucose tolerance and insulin secretion in rats exposed to a high-fat diet (Cani et al., 2007). Propionic acid, a key gut-produced substance, was found to directly benefit adipose tissue by reducing inflammation linked to obesity and increasing both glucose absorption and fat production. Butyric acid, known for being a significant energy source for the colonic epithelium, demonstrated a positive effect on diabetes and insulin resistance (Guo et al., 2022, Wu et al., 2022).

These SCFA productions during fecal fermentation comply with the results of the prebiotic effect on selected probiotics (Fig. S4), where: EPS-LB13 and EPS-MLB10 utilized when used as carbohydrate source that were replaced and utilized, which was the case in previous studies (Fu et al., 2018, Xie et al., 2019). Both EPSs are comparable to the commercial prebiotic GOS-P in terms of stimulating the SCFA production by the gut microbiota suggesting EPS-LB13 and EPS-MLB10 can be a good candidate for a prebiotic activity in health and food industries.

#### 3.5.2 Effect of EPS-LB13 and EPS-MLB10 on microbial and functional profile

**Fig. 3:**
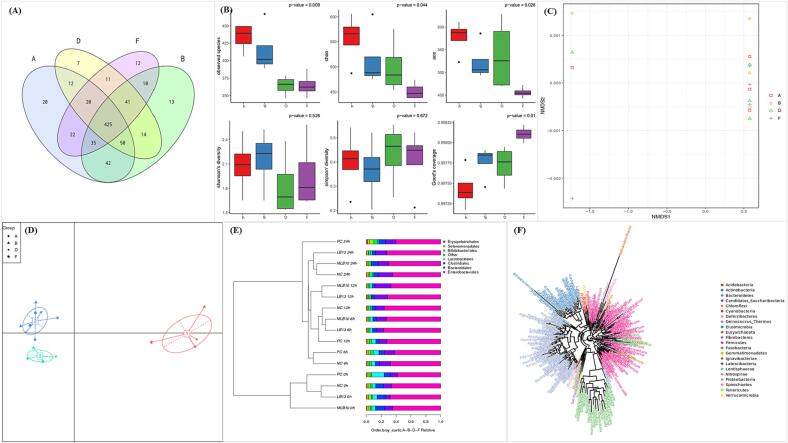
Effect of EPS on gut microbiome composition during fecal fermentation. Venn Diagram (A), Box Plot of Different Indices of Alpha Diversity (B), NMDS plot (C), PCoA plot (D), Combination Graph of UPGMA Cluster Tree and Order Abundance Bar plot (E), and Species Phylogenetic Analysis (F) of the Sample Groups. Where A, B, D, and F are the Sample Groups: (A) Blank (Negative Control), (B) GOS-P (Positive Control), (D) EPS-LB13, and (F) EPS-MLB10.

The effect of purified EPSs on the gut microbiota was assessed by 16S-rRNA gene sequencing using the Illumina MiSeq platform and the V3-V4 region of the 16S-rRNA gene. A Venn diagram (Fig. 3a) shows the number of shared and unique OTUs in the four groups: blank, GOS-P, EPS-LB13 and EPS-MLB10. The results showed that during *in vitro* fecal fermentation, a total of 425 OTUs were shared among the groups, of which 41 OTUs were shared among the EPS-LB13, EPS-MLB10 and GOS-P groups, which shows the similarity of gut microbiota among the EPSs and GOS-P groups. The EPS-LB13 and EPS-MLB10 groups had the least unique OTUs 7 and 12, respectively, compared to the blank group which had 20 OTUs, suggesting that they stimulated the growth of specific microbiota in the gut. Furthermore, multiple response permutation procedure (MRPP) and analysis of similarities (ANOSIM) of the OTU data showed no significant (P > 0.05) difference among the groups with −0.11 delta value and −0.07 R value (data not shown), indicating that the effect of EPSs on the gut microbiota was not strong enough to alter their diversity at the OTU level.

The diversity analysis included alpha diversity and the beta diversity of the four groups during fecal fermentation. The alpha diversity was determined by plotting rarefaction curves and boxplots for six diversity metrics: observed species, Chao, ACE, Shannon, Simpson, and Good's coverage indices. These metrics reflect different aspects of the microbial community structure, such as species richness (observed species, Chao, ACE), diversity and evenness (Shannon, Simpson), and reliability of sample sequencing (Good's coverage) (Yi et al., 2022). All the rarefaction curves (Fig. S5) showed similar shapes for all the groups, which indicated that each group had similar patterns of diversity and sampling completeness. However, there were some differences in the levels of diversity among the groups, as shown by the box plot (Fig. 3b). The EPS-LB13 group showed the lowest diversity, followed by EPS-MLB10 and GOS-P, suggesting the selective growth of specific species. Whereas beta diversity was used to evaluate differences of sample groups in species complexity. The Non-metric Multi-dimensional Scaling (NMDS) plot showed that the groups from time 0 h were clearly distinct from the other time points on the NMDS plot (Fig. 3c), reflecting the initial diversity and composition of the microbial communities. The treatments had a strong effect on the microbial communities, causing them to converge to a similar state by 24 h (Fig. 3c). The principle coordinate analysis (PCoA) diagram of enterotypes suggests the same as the enterotype 1 was distinct from the other two enterotypes (Fig. 3d) which was from time 0 h, suggesting that the groups induced a shift in the enterotypes over time, which may be due to the metabolic functions. Furthermore, the Kruskal-Walli’s test showed that most of the orders do not show a significant difference in abundance across groups, as their p-values are high which was seen in the alpha diversity. Bray-Curtis and the relative abundance studies (Fig. 3e), shows that among the orders, Enteriobacteriales were most abundant throughout the fermentation in all the groups which mostly consist of the opportunistic bacteria, Bacteriodales. Clostridales and Lactobacillales were other orders which were abundant and are known to be responsible for the SCFA production (Xie et al., 2019, Yi et al., 2022). The species phylogenetic tree agrees with the abundance results of the orders as the species abundancy was mainly from the phylum Firmicutes followed by Proteobacteria, Bacteroidetes, and Actinobacteria (Fig. 3f). In conclusion, the OTU abundance data and the alpha and beta diversity data showed that there was no significant difference in the gut microbiota of the blank and the treated samples. However, some specific bacteria were promoted in the EPS-LB13, EPS-MLB10 and GOS-P groups which can be confirmed using function prediction.

**Fig. 4:**
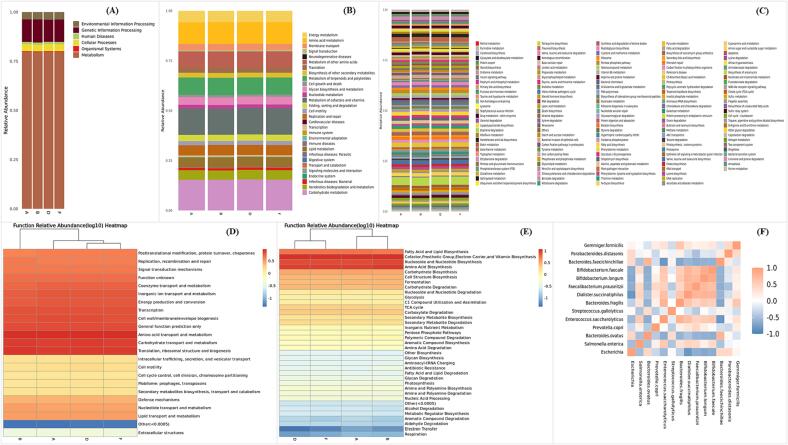
Effect of EPSs on different microbial functions during fecal fermentation. Histograms of KEGG Pathways abundance at Levels 1–3 (A-C), Heatmap of COG Pathways (D), Heatmap of MetaCyc Pathways (E), and Species Spearman Coefficients Analysis of OTUs in Each Sample Group (F). Where A, B, D and F are the Sample Groups: (A) Blank (Negative Control), (B) GOS-P (Positive Control), (D) EPS-LB13, and (F) EPS-MLB10.

The microbial functions, including the abundance of KEGG, COG, and MetaCyc metabolic pathways in the microbial community based on marker gene sequencing profiles were predicted by PICRUSt2 (Phylogenetic Investigation of Communities by Reconstruction of Unobserved States). The KEGG data showed the relative abundance of different metabolic pathways during fecal fermentation in the four groups and KEGG level 1 (Fig. 4a) showed the highest abundance of metabolism pathways in all the groups which was due to digestion (Teame et al., 2020). KEGG level 2 (Fig. 4b) showed that among the metabolism category, the carbohydrate metabolism, amino acid metabolism, metabolism of cofactors and vitamins, and biosynthesis of other secondary metabolites pathways had the highest abundance in all groups. KEGG level 3 (Fig. 4c) showed slight differences in the metabolism pathways within the groups. Among the metabolism, EPS-LB13 had the highest abundance of carbohydrate metabolism which includes pentose phosphate pathway, carbon fixation pathways in prokaryotes, and photosynthesis sub-pathways and in the amino acid metabolism whereas EPS-MLB10 group had the highest abundance of cysteine and methionine metabolism, glycine, serine and threonine metabolism, and phenylalanine, tyrosine and tryptophan biosynthesis sub-pathways. The COG heatmap complemented the KEGG results showing relative abundance of different functional categories during fecal fermentation in the four groups (Fig. 4d). The most abundant functions (Fig. 4d) in the microbial community included carbohydrates and amino acids transport and metabolism in all the four groups which plays major role in the digestion of undigested carbohydrates and proteins to short-chain fatty acids. Other abundant functions (Fig. 4d) included translation, ribosome structure and biogenesis which are essential for cellular growth, differentiation, and adaptation and secondary metabolites and coenzyme transport and metabolism which are known to produce precursors for SCFA production and maintain metabolic homeostasis. Furthermore, the MetaCyc heatmap (Fig. 4e) confirmed the most abundant pathways in the microbial community included glycolysis, pyruvate metabolism, TCA cycle, and fermentation, which are involved in the breakdown of carbohydrates and production of SCFAs (De Vos, Tilg, Van Hul, & Cani, 2022). It also shows that the inclusion of amino acid biosynthesis and degradation, which are important for nitrogen metabolism and protein synthesis, and vitamin biosynthesis, complementing the biosynthesis of secondary metabolites (Manor et al., 2020). EPS-LB13 group had the highest abundance of pathways related to glycolysis, pyruvate metabolism, TCA cycle, and fermentation, indicating a high capacity for carbohydrate utilization and SCFA production which was observed in (Fig. 4e). These results suggest that the different EPSs modulate the metabolic functions of the fecal microbiota in different ways, which may have implications for the host health.

#### 3.5.3 Correlation analysis

The Spearman correlation and network analysis were used to explore the associations among the different species in the fecal microbiota of the four groups. The Spearman correlation matrix (Fig. 4f) showed the pairwise correlation coefficients between the species during fecal fermentation. For example, *Bifidobacterium longum* has strong positive correlations (*P < 0.05*) with *Dialister succinatiphilus*, *Faecalibacterium prausnitzii*, *Bifidobacterium faecale*, and *Gemmiger formicilis*, and moderate positive correlations (*P < 0.05*) with *Bacteroides fragilis* and *Streptococcus gallolyticus*, which are known to produce SCFAs that have anti-inflammatory and immunomodulatory effects (De Vos et al., 2022). Whereas *Escherichia* has strong positive correlations (*P < 0.05*) with *Salmonella enterica*, *Bacteroides faecichinchillae*, and *Parabacteroides distasonis*, and strong and significant negative correlations (*P < 0.05*) with *Dialister succinatiphilus*, *Bifidobacterium longum*, *Bifidobacterium faecale*, and *Faecalibacterium prausnitzii*, which are opportunistic pathogens that can cause intestinal infection and inflammation (De Vos et al., 2022). Additionally, the network analysis (Fig. S6a) showed the positive correlation (*P < 0.05*) between *Bacteroides ovatus* and *Bacteroides faecichinchillae*, and *Bacteroides ovatus* had a strong negative correlation (*P < 0.05*) with *Enterococcus saccharolyticus* in most sample groups. Moreover, the relative abundance of phyla during fermentation (Fig. S6b) shows the abundance of Proteobacteria, Bacteroidetes, Firmicutes, and Acinetobacter from which the species belong to in the Spearman correlation and network analysis data. These phyla mostly digest the complex dietary fibers to produce beneficial metabolites, such as SCFAs, antibiotics, and siderophores, which may modulate host immunity, inflammation, and energy homeostasis (De Vos et al., 2022, Manor et al., 2020). These results suggest that there are complex ecological interactions and metabolic dependencies among the bacterial species in different sample groups during fermentation.

### 3.6 EPS impact on rheology of fermented skimmed milk

**Fig. 5:**
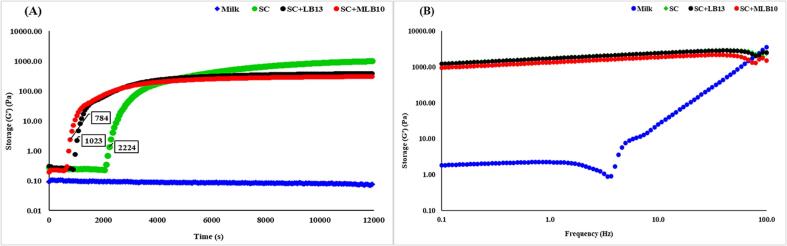
Fermented bovine milk storage modulus during (A) Time Sweep and (B) Frequency Sweep of Yo-Flex Chr. Hansen culture (SC), SC + *E. faecium* MW725386 (EPS-LB13), and SC + and *Streptococcus thermophilus* MW725391(EPS-MLB10).

The viscoelastic properties of skimmed bovine milk fermented with different strains were evaluated by measuring the storage modulus (G’) and loss modulus (G”) using time sweep and frequency sweep tests. The storage modulus (G’) reflects the elastic behavior of the material, while the loss modulus (G”) reflects the viscous behavior (Tiwari et al., 2021). The G’ values of all samples increased with time and the milk fermented with starter culture alone showed slightly higher G’ value. The gelation started earliest in SC + MLB10 followed by SC + LB13 and SC (Fig. 5a) at 784, 1023 and 2224s, respectively, indicating that the addition of EPS-producing bacteria affected gel formation time. The G” values showed a similar trend (Fig. S7a) to those of G’ in time sweep, but was lower than G’ values suggesting that the skimmed milk reached a steady state of viscous behavior and energy dissipation during fermentation (Tiwari et al., 2021). After the fermentation of the skimmed milk, G’ values (Fig. 5b) increased at frequencies of 42.99, 39.81, and 34.15 Hz in the frequency sweep for the milk fermented with SC, SC + LB13 and SC + MLB10, respectively, then slight decrease in the G’ values were observed, and this may be due to a decrease in elasticity at high frequencies. However, G” values (Fig. S7b) increased as frequency increased, which indicates that the gels formed were viscous and resistant to deformation at high frequencies. These rheological properties of skimmed bovine milk, when fermented with EPS-producing bacteria, have hastened the gelation process, resulting in the faster formation of gel networks with solid-like properties. The observed differences in storage and loss moduli values imply that the type of starter cultures and EPS-producing bacteria can significantly influence the rheological properties of milk fermentation as observed in previous studies (Ali et al., 2023, Ayyash et al., 2020). Nonetheless, a thorough evaluation of the sensory attributes, such as mouth feel, thickness, creaminess, and firmness, of this fermented low-fat bovine milk has to be investigated to fully understand the impact of EPS.

## 4 Conclusions

The high molecular weight heteropolysaccharides produced by Enterococcus faecium MW725386 and Streptococcus thermophilus MW725391, isolated from Labneh, demonstrated great potential as prebiotics that support the growth of probiotics and also revealed good bioactivities, including antidiabetic, anticancer, antimicrobial. It was also observed that the EPSs had a slightly positive impact on the gut microbiota during *in vitro* fecal fermentation, producing SCFA, which can regulate intestinal homeostasis. Additionally, EPS-producing bacteria positively impacted the gelation time and stiffness of the gel formed during bovine skimmed milk fermentation, which suggests that these EPSs could be used for the fermentation of camel milk. Furthermore, extensive research *in vivo* studies and sensory evaluations of fermented milk have to be conducted to consider these EPSs in the pharmaceutical and dairy industries, respectively.

## CRediT authorship contribution statement

**Mohammed Tarique:** Data curation, Formal analysis, Investigation, Writing – original draft. **Abdelmoneim H. Ali:** Writing – original draft, Writing – review & editing. **Jaleel Kizhakkayil:** Investigation. **Shao-Quan Liu:** Writing – review & editing. **Fatih Oz:** Writing – review & editing. **Enes Dertli:** Validation, Visualization, Writing – review & editing. **Afaf Kamal-Eldin:** Validation, Visualization, Writing – review & editing. **Mutamed Ayyash:** Conceptualization, Data curation, Formal analysis, Funding acquisition, Project administration, Resources, Supervision, Validation, Visualization, Writing – review & editing.

## Declaration of competing interest

The authors declare the following financial interests/personal relationships which may be considered as potential competing interests: None.

## Data Availability

Data will be made available on request.

## References

[b0005] Abarquero D., Renes E., Fresno J.M., Tornadijo M.E. (2022). Study of exopolysaccharides from lactic acid bacteria and their industrial applications: A review. International Journal of Food Science & Technology.

[b0010] Abdalla A.K., Ayyash M.M., Olaimat A.N., Osaili T.M., Al-Nabulsi A.A., Shah N.P., Holley R. (2021). Exopolysaccharides as Antimicrobial Agents: Mechanism and Spectrum of Activity. Frontiers in Microbiology.

[b0015] Adebayo-Tayo B., Ishola R., Oyewunmi T. (2018). Characterization, antioxidant and immunomodulatory potential on exopolysaccharide produced by wild type and mutant Weissella confusa strains. Biotechnology Reports.

[b0020] Al-Nabulsi A.A., Jaradat Z.W., Al Qudsi F.R., Elsalem L., Osaili T.M., Olaimat A.N., Esposito G., Liu S.-Q., Ayyash M.M. (2022). Characterization and bioactive properties of exopolysaccharides produced by Streptococcus thermophilus and Lactobacillus bulgaricus isolated from labaneh. LWT.

[b0025] Ali A.H., Bamigbade G., Tarique M., Esposito G., Obaid R., Abu-Jdayil B., Ayyash M. (2023). Physicochemical, rheological, and bioactive properties of exopolysaccharide produced by a potential probiotic Enterococcus faecalis 84B. International Journal of Biological Macromolecules.

[b0030] Andrew M., Jayaraman G. (2020). Structural features of microbial exopolysaccharides in relation to their antioxidant activity. Carbohydrate Research.

[b0035] Angelin J., Kavitha M. (2020). Exopolysaccharides from probiotic bacteria and their health potential. International Journal of Biological Macromolecules.

[b0040] Ayyash M., Al-Nuaimi A.K., Al-Mahadin S., Liu S.-Q. (2018). In vitro investigation of anticancer and ACE-inhibiting activity, α-amylase and α-glucosidase inhibition, and antioxidant activity of camel milk fermented with camel milk probiotic: A comparative study with fermented bovine milk. Food Chemistry.

[b0045] Ayyash M., Stathopoulos C., Abu-Jdayil B., Esposito G., Baig M., Turner M.S., Baba A.S., Apostolopoulos V., Al-Nabulsi A., Osaili T. (2020). Exopolysaccharide produced by potential probiotic Enterococcus faecium MS79: Characterization, bioactivities and rheological properties influenced by salt and pH. LWT.

[b0050] Bello F.D., Walter J., Hertel C., Hammes W.P. (2001). In vitro study of Prebiotic Properties of Levan-type Exopolysaccharides from Lactobacilli and Non-digestible Carbohydrates Using Denaturing Gradient Gel Electrophoresis. Systematic and Applied Microbiology.

[b0055] Bhat B., Bajaj B.K. (2018). Hypocholesterolemic and bioactive potential of exopolysaccharide from a probiotic Enterococcus faecium K1 isolated from kalarei. Bioresource Technology.

[b0060] Cani P.D., Neyrinck A.M., Fava F., Knauf C., Burcelin R.G., Tuohy K.M., Gibson G.R., Delzenne N.M. (2007). Selective increases of bifidobacteria in gut microflora improve high-fat-diet-induced diabetes in mice through a mechanism associated with endotoxaemia. Diabetologia.

[b0065] Choudhuri I., Khanra K., Pariya P., Maity G.N., Mondal S., Pati B.R., Bhattacharyya N. (2020). Structural Characterization of an Exopolysaccharide Isolated from Enterococcus faecalis, and Study on its Antioxidant Activity, and Cytotoxicity Against HeLa Cells. Current Microbiology.

[b0070] De Vos W.M., Tilg H., Van Hul M., Cani P.D. (2022). Gut microbiome and health: Mechanistic insights. Gut.

[b0075] Ding Y., Yan Y., Peng Y., Chen D., Mi J., Lu L., Luo Q., Li X., Zeng X., Cao Y. (2019). In vitro digestion under simulated saliva, gastric and small intestinal conditions and fermentation by human gut microbiota of polysaccharides from the fruits of Lycium barbarum. International Journal of Biological Macromolecules.

[b0080] Dobrowolska-Iwanek J., Lauterbach R., Huras H., Paśko P., Prochownik E., Woźniakiewicz M., Chrząszcz S., Zagrodzki P. (2020). HPLC-DAD method for the quantitative determination of short-chain fatty acids in meconium samples. Microchemical Journal.

[b0085] DuBois M., Gilles K.A., Hamilton J.K., Rebers P.A., Smith F. (1956). Colorimetric Method for Determination of Sugars and Related Substances. Analytical Chemistry.

[b0090] Fu X., Cao C., Ren B., Zhang B., Huang Q., Li C. (2018). Structural characterization and in vitro fermentation of a novel polysaccharide from Sargassum thunbergii and its impact on gut microbiota. Carbohydrate Polymers.

[b0095] Guo D., Lei J., He C., Peng Z., Liu R., Pan X., Meng J., Feng C., Xu L., Cheng Y., Chang M., Geng X. (2022). In vitro digestion and fermentation by human fecal microbiota of polysaccharides from Clitocybe squamulose. International Journal of Biological Macromolecules.

[b0100] Hu S.-M., Zhou J.-M., Zhou Q.-Q., Li P., Xie Y.-Y., Zhou T., Gu Q. (2021). Purification, characterization and biological activities of exopolysaccharides from Lactobacillus rhamnosus ZFM231 isolated from milk. LWT.

[b0105] Jia K., Tao X., Liu Z., Zhan H., He W., Zhang Z., Zeng Z., Wei H. (2019). Characterization of novel exopolysaccharide of Enterococcus faecium WEFA23 from infant and demonstration of its in vitro biological properties. International Journal of Biological Macromolecules.

[b0110] Leivers S., Hidalgo-Cantabrana C., Robinson G., Margolles A., Ruas-Madiedo P., Laws A.P. (2011). Structure of the high molecular weight exopolysaccharide produced by Bifidobacterium animalis subsp. lactis IPLA-R1 and sequence analysis of its putative eps cluster. Carbohydrate Research.

[b0115] Li S., Shah N.P. (2016). Characterization, Anti-Inflammatory and Antiproliferative Activities of Natural and Sulfonated Exo-Polysaccharides from *Streptococcus thermophilus* ASCC 1275: Bioactivities of exo-polysaccharides…. Journal of Food Science.

[b0120] Lobo R.E., Gómez M.I., Font De Valdez G., Torino M.I. (2019). Physicochemical and antioxidant properties of a gastroprotective exopolysaccharide produced by Streptococcus thermophilus CRL1190. Food Hydrocolloids.

[b0125] Magoč T., Salzberg S.L. (2011). FLASH: Fast length adjustment of short reads to improve genome assemblies. Bioinformatics.

[b0130] Manor O., Dai C.L., Kornilov S.A., Smith B., Price N.D., Lovejoy J.C., Gibbons S.M., Magis A.T. (2020). Health and disease markers correlate with gut microbiome composition across thousands of people. Nature Communications.

[b0135] Pan L., Han Y., Zhou Z. (2020). In vitro prebiotic activities of exopolysaccharide from Leuconostoc pseudomesenteroides XG5 and its effect on the gut microbiota of mice. Journal of Functional Foods.

[b0140] Sharma S.K. (2015). Optimized Extraction and Antioxidant Activities of Polysaccharides from Two Entomogenous Fungi. Journal of Bioanalysis & Biomedicine.

[b0145] Sirin S., Aslim B. (2020). Characterization of lactic acid bacteria derived exopolysaccharides for use as a defined neuroprotective agent against amyloid beta1–42-induced apoptosis in SH-SY5Y cells. Scientific Reports.

[b0150] Siswoyo T.A., Arum L.S., Sanjaya B.R.L., Aisyah Z.S. (2021). The growth responses and antioxidant capabilities of melinjo (Gnetum gnemon L.) in different durations of drought stress. Annals of Agricultural Sciences.

[b0155] Tarique M., Abdalla A., Masad R., Al-Sbiei A., Kizhakkayil J., Osaili T., Olaimat A., Liu S.-Q., Fernandez-Cabezudo M., al-Ramadi B., Ayyash M. (2022). Potential probiotics and postbiotic characteristics including immunomodulatory effects of lactic acid bacteria isolated from traditional yogurt-like products. LWT.

[b0160] Teame, T., Wang, A., Xie, M., Zhang, Z., Yang, Y., Ding, Q., Gao, C., Olsen, R. E., Ran, C., & Zhou, Z. (2020). Paraprobiotics and Postbiotics of Probiotic Lactobacilli, Their Positive Effects on the Host and Action Mechanisms: A Review. *Frontiers in Nutrition*, *7*, 570344. doi: 10.3389/fnut.2020.570344. 10.3389/fnut.2020.570344 PMC7642493 33195367

[b0165] Tiwari S., Kavitake D., Devi P.B., Halady Shetty P. (2021). Bacterial exopolysaccharides for improvement of technological, functional and rheological properties of yoghurt. International Journal of Biological Macromolecules.

[b0170] Vojvodić Cebin A., Komes D., Ralet M.-C. (2022). Development and Validation of HPLC-DAD Method with Pre-Column PMP Derivatization for Monomeric Profile Analysis of Polysaccharides from Agro-Industrial Wastes. Polymers.

[b0175] Wang J., Ji H.F., Wang S.X., Zhang D.Y., Liu H., Shan D.C., Wang Y.M. (2012). Lactobacillus plantarum ZLP001: In vitro Assessment of Antioxidant Capacity and Effect on Growth Performance and Antioxidant Status in Weaning Piglets. Asian-Australasian Journal of Animal Sciences.

[b0180] Wang J., Zhao X., Yang Y., Zhao A., Yang Z. (2015). Characterization and bioactivities of an exopolysaccharide produced by Lactobacillus plantarum YW32. International Journal of Biological Macromolecules.

[b0185] Wang K.W., Sheng X.Y., Wu B., Wang H., Chen J.B., Wang S.W. (2023). Structure characterization of novel heteropolysaccharides from Pteridium revolutum with antioxidant and antiglycated activities. Food Chem X.

[b0190] Wu D.-T., An L.-Y., Liu W., Hu Y.-C., Wang S.-P., Zou L. (2022). In vitro fecal fermentation properties of polysaccharides from Tremella fuciformis and related modulation effects on gut microbiota. Food Research International.

[b0195] Xie Z., Wang S., Wang Z., Fu X., Huang Q., Yuan Y., Wang K., Zhang B. (2019). In vitro fecal fermentation of propionylated high-amylose maize starch and its impact on gut microbiota. Carbohydrate Polymers.

[b0200] Xu Z., Li X., Tian X., Yang S., Li Y., Li Z., Guo T., Kong J. (2023). Characterization of the antioxidant activities of the exopolysaccharides produced by Streptococcus thermophilus CGMCC 7.179. LWT.

[b0205] Yang S., Xu X., Peng Q., Ma L., Qiao Y., Shi B. (2023). Exopolysaccharides from lactic acid bacteria, as an alternative to antibiotics, on regulation of intestinal health and the immune system. Animal Nutrition.

[b0210] Yi C., Xu L., Luo C., He H., Ai X., Zhu H. (2022). In vitro digestion, fecal fermentation, and gut bacteria regulation of brown rice gel prepared from rice slurry backfilled with rice bran. Food Hydrocolloids.

[b0215] Yilmaz M.T., İspirli H., Taylan O., Taşdemir V., Sagdic O., Dertli E. (2022). Characterisation and functional roles of a highly branched dextran produced by a bee pollen isolate Leuconostoc mesenteroides BI-20. Food Bioscience.

[b0220] Yılmaz T., Şimşek Ö. (2020). Potential Health Benefits of Ropy Exopolysaccharides Produced by Lactobacillus plantarum. Molecules.

[b0225] Zhang J., Cao Y., Wang J., Guo X., Zheng Y., Zhao W., Mei X., Guo T., Yang Z. (2016). Physicochemical characteristics and bioactivities of the exopolysaccharide and its sulphated polymer from Streptococcus thermophilus GST-6. Carbohydrate Polymers.

[b0230] Zhu Y., Wang X., Pan W., Shen X., He Y., Yin H., Zhou K., Zou L., Chen S., Liu S. (2019). Exopolysaccharides produced by yogurt-texture improving Lactobacillus plantarum RS20D and the immunoregulatory activity. International Journal of Biological Macromolecules.

