# Supplementary material for: Exopolysaccharides from Enterococcus faecium and Streptococcus thermophilus: Bioactivities, gut microbiome effects, and fermented milk rheology
Source: Food Chem X. 2023 Dec 15;21:101073. doi: 10.1016/j.fochx.2023.101073 (PMC10792183; doi:10.1016/j.fochx.2023.101073)
Supplement: Supplementary data 1 [file mmc1.pdf]

Supplementary Materials

Exopolysaccharides from *Enterococcus faecium* and *Streptococcus thermophilus*: Bioactivities, Gut Microbiome Effects, and Fermented Milk Rheology

Mohammed Tarique[1], Abdelmoneim H. Ali[2], Jaleel Kizhakkayil[3], Shao-Quan Liu[4], Fatih Oz[5], Enes Dertli[6], Afaf Kamal-Eldin[1], Mutamed Ayyash[1*]

[1] Department of Food Science, College of Agriculture and Veterinary Medicine, United Arab Emirates University (UAEU), Al Ain, UAE

[2] Department of Food Science, Faculty of Agriculture, Zagazig University, Zagazig 44511, Egypt

[3] Department of Nutrition and Health Sciences, College of Medicine and Health Sciences, United Arab Emirates University (UAEU), Al Ain, UAE

[4] Department of Food Science and Technology, Faculty of Science, National University of Singapore, Science Drive 2, Singapore 117542

[5] Department of Food Engineering, Faculty of Agriculture, Ataturk University, Erzurum 25240, Turkey.

[6] Department of Food Engineering, Faculty of Chemical and Metallurgical Engineering, Yildiz Technical University, İstanbul, Turkey

*Corresponding author:

Mutamed Ayyash

Email: mutamed.ayyash@uaeu.ac.ae

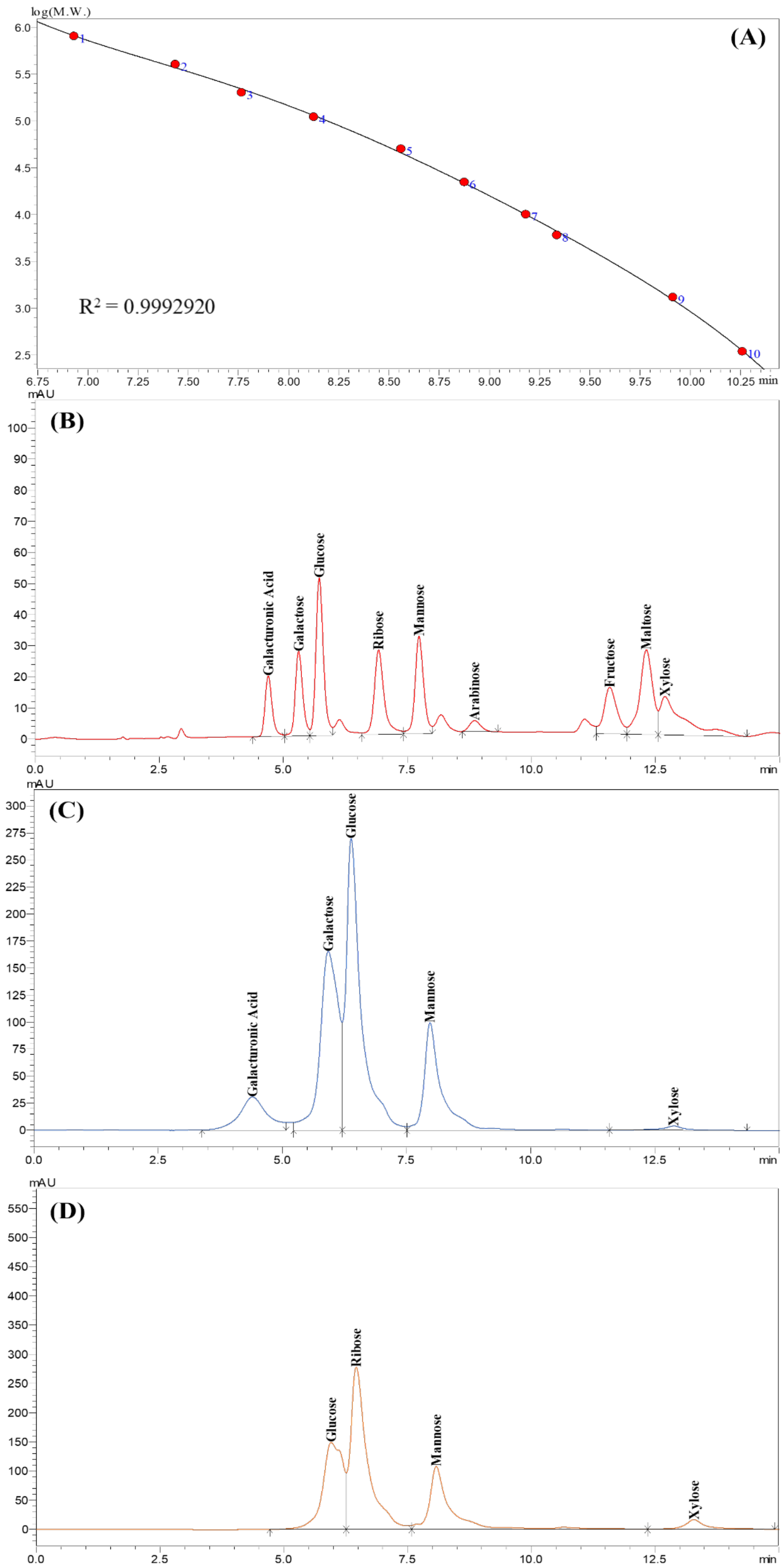

Figure S1: GPC Standard Curve Pullulan Standards (800-0.342 kDa) (A), HPLC Chromatograms of Standards (B), Hydrolyzed EPS-LB13 (C), and Hydrolyzed EPS-MLB10 (D) of PMP-Derivatized Monosaccharides.

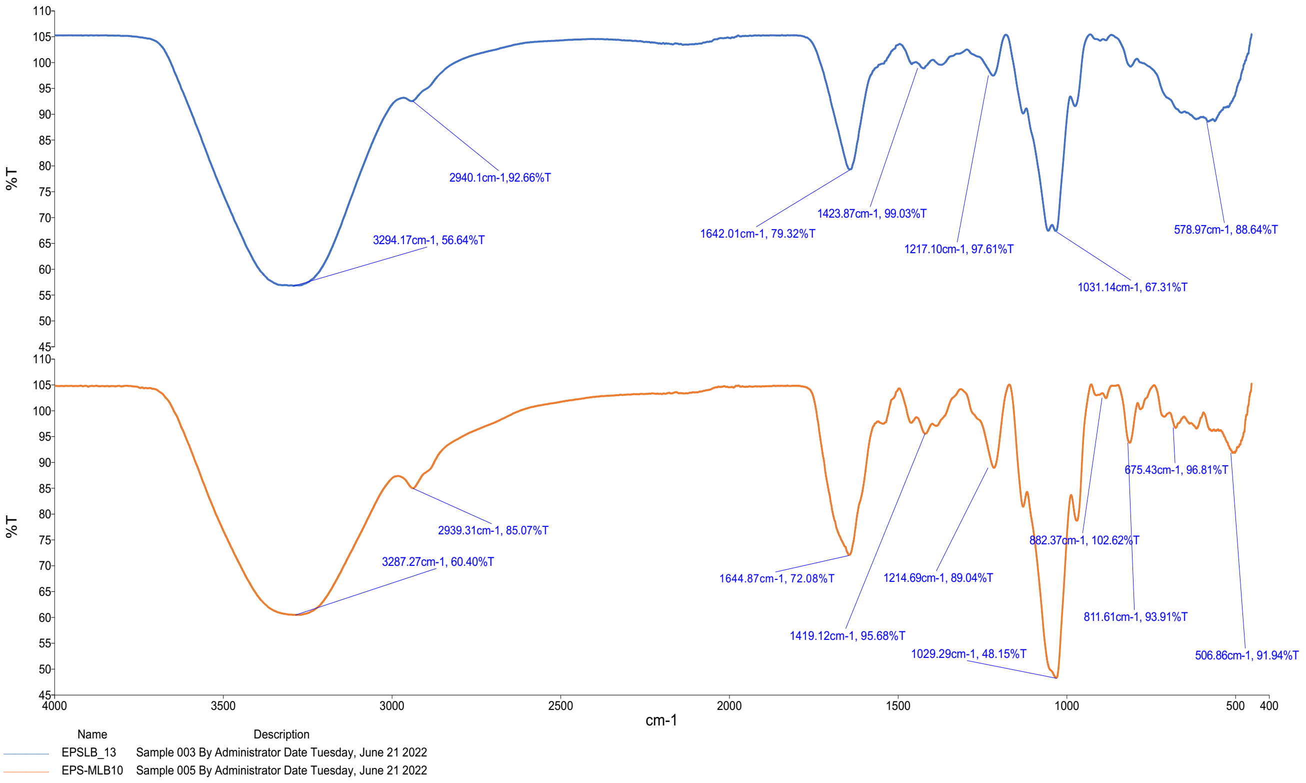

Figure S2: FTIR Spectrum of EPS-LB13 and EPS-MLB10.

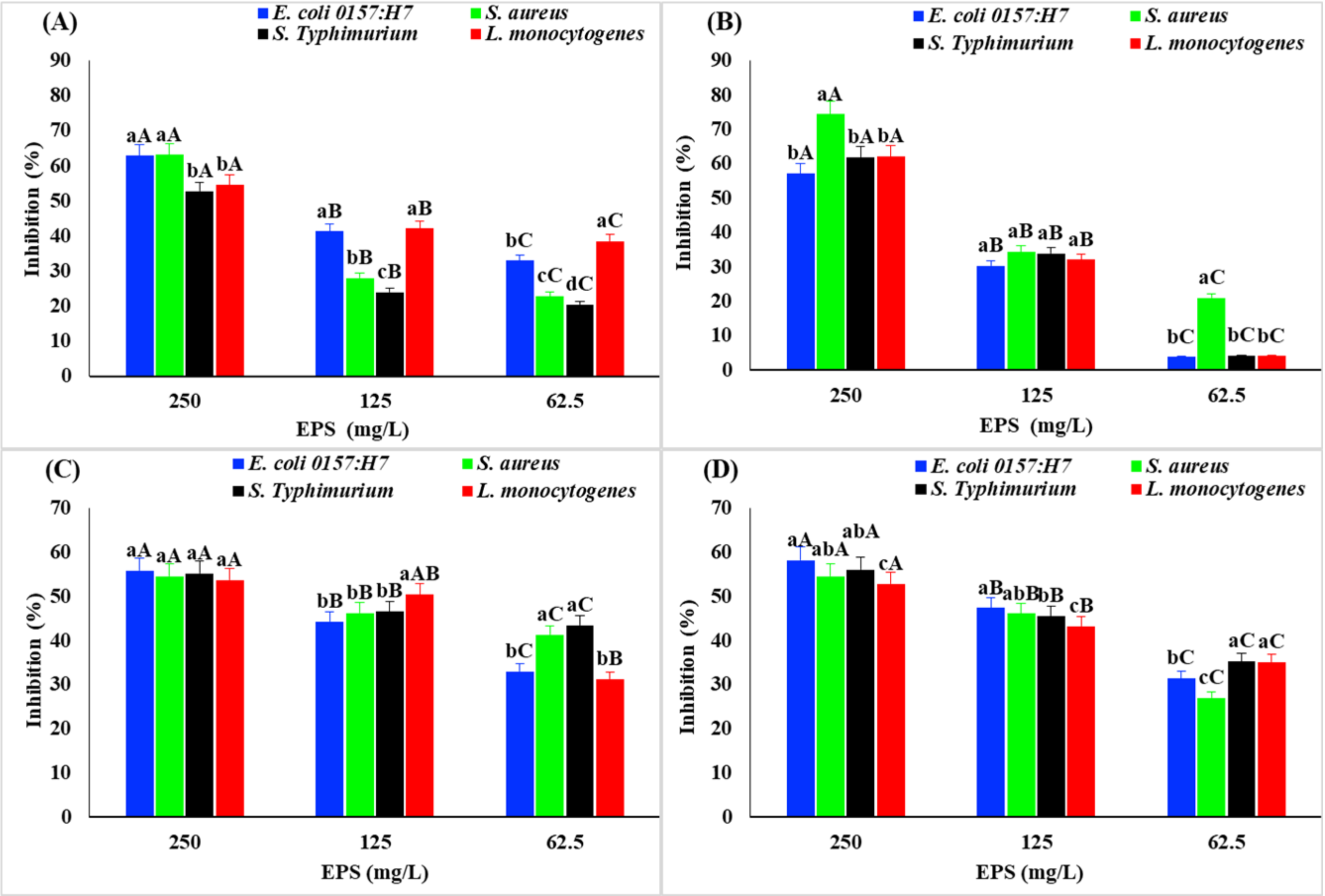

Figure S3: Minimum Inhibitory Concentration of EPS-LB13 (A), and EPS-MLB10 (B), Biofilm Inhibitory Activity of EPS-LB13 (C), and EPS-MLB10 (D). Bars are the mean values ± standard deviations (error bars). [a-d] Means with different lowercase letters at same parameter differed significantly ($P < 0.05$). [A-D] Means with different uppercase letters differed significantly ($P < 0.05$).

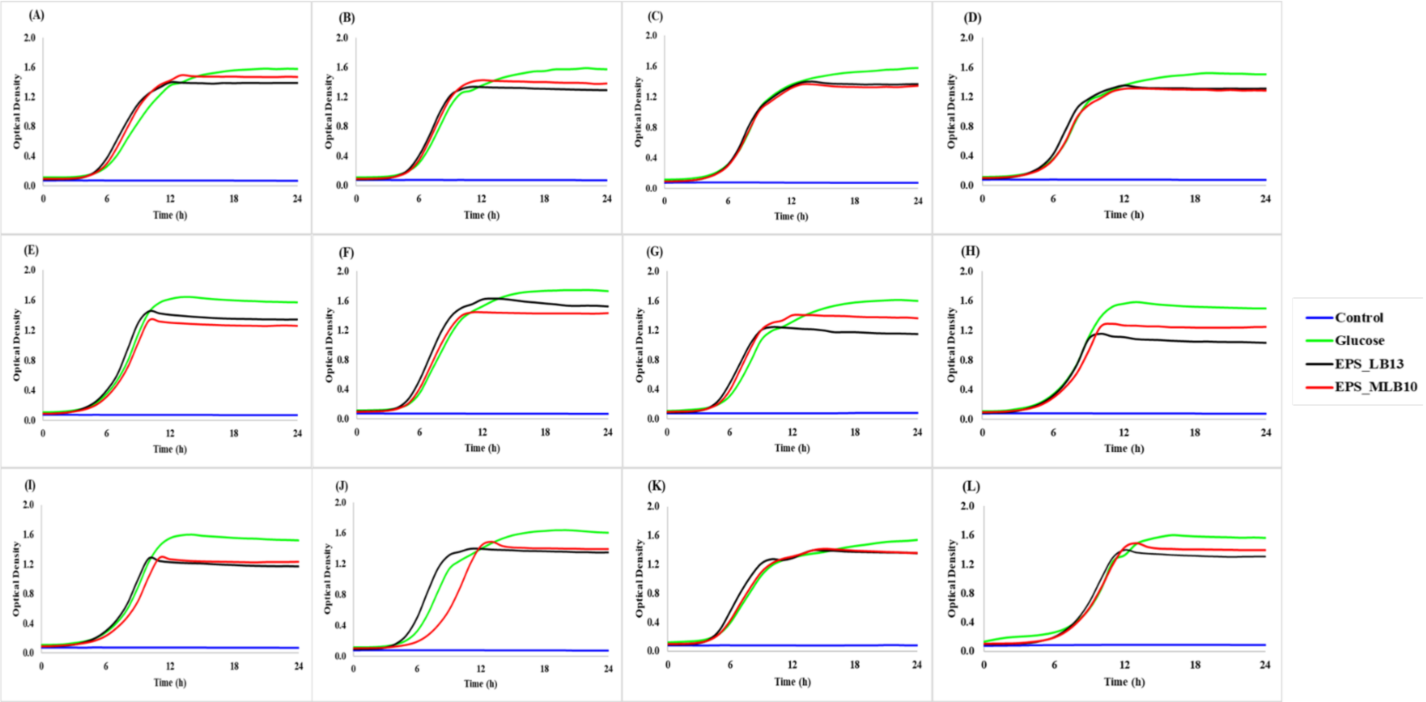

Figure S4: Growth curve of probiotic strains *L. acidophilus* (A), *B. longum* subsp. *longum* (B), *L. delbrueckii subsp. delbrueckii* (C), *L. delbrueckii* subsp. *lactis* (D), *L. rhamnosus* (E), *L. paracasei* subsp. *paracasei* (F), *L. plantarum* (G), *L .paracasei* subsp. *tolerans* (H), *L. gasseri* (I), *L. delbrueckii* subsp. *bulgaricus* (J), *B. breve* (K), and *B. animalis* subsp. *lactis* (L) with EPS-LB13, EPS-MLB10, and Glucose. Control was with no sugar.

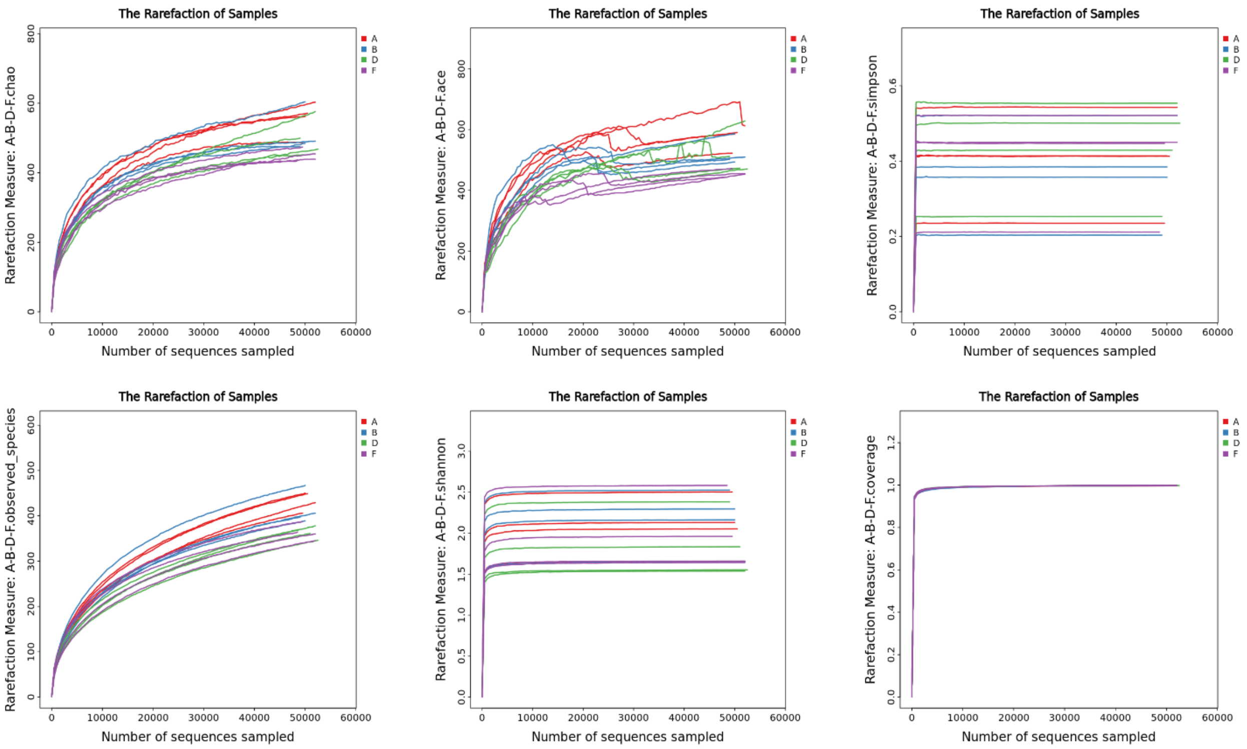

Figure S5: Alpha diversity rarefaction curves of different indices during *In vitro* fecal fermentation. Where A, B, D, and F are the Sample Groups: (A) Blank (negative control), (B) GOS-P (positive control), (D) EPS-LB13, and (F) EPS-MLB10.

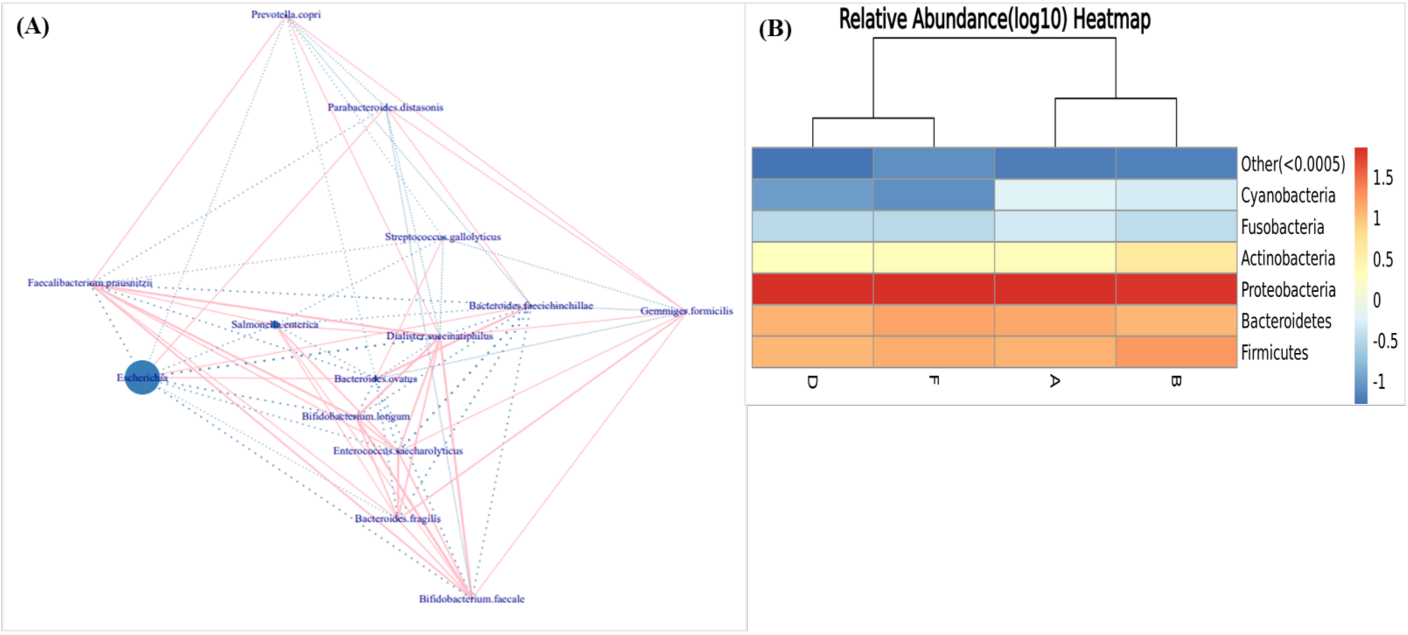

Figure S6: Species network and phylum heatmap of *in vitro* fecal fermentation. (A) Species Network, where Nodes Represent Species, Node Area Represents Average Relative Abundance, Ligand Color Represents Correlation Sign, and Ligand Thickness Represents Correlation Level. (B) Phylum Heatmap, where Horizontal Clusters Represent the Similarity of Species among Samples and Relative Abundance Values are Log-Transformed. Where A, B, D, and F are the Sample Groups: (A) Blank (negative control), (B) GOS-P (positive control), (D) EPS-LB13, and (F) EPS-MLB10.

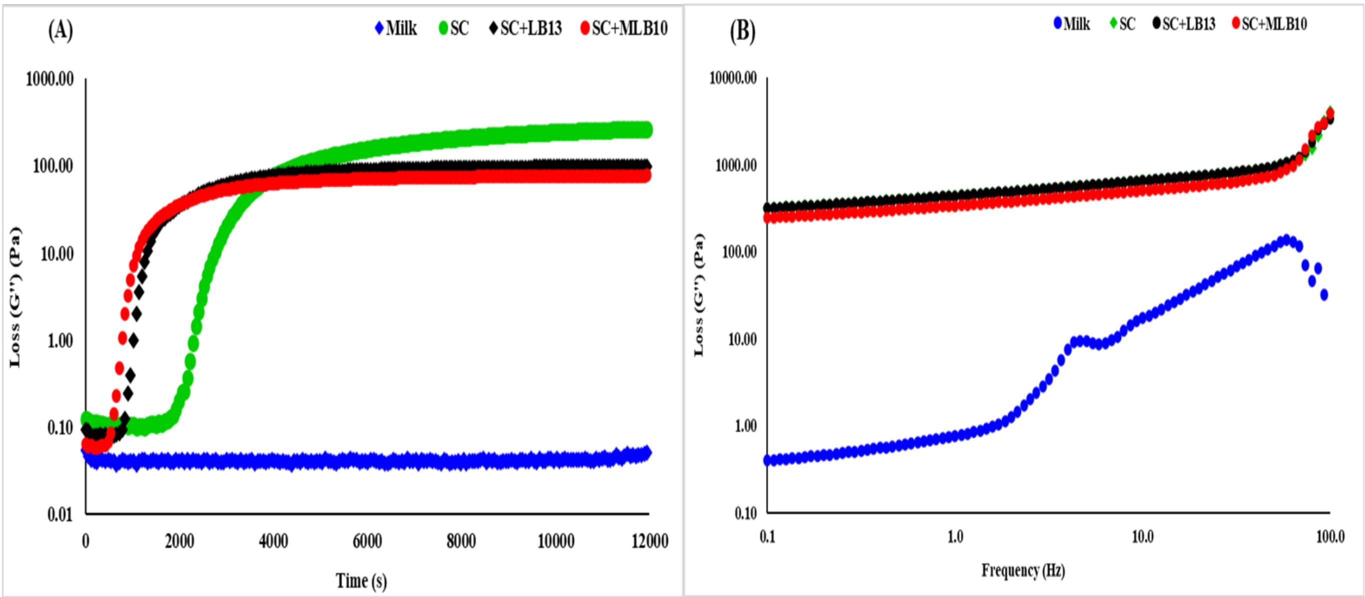

Figure S7: Fermented bovine milk loss modulus during (A) Time Sweep and (B) Frequency Sweep of Yo-Flex Chr. Hansen culture (SC), SC+ *Enterococcus faecium* MW725386 (EPS-LB13), and SC+ *Streptococcus thermophilus* MW725391 (EPS-MLB10).
